# Novel Insights into miRNA Regulation of Storage Protein Biosynthesis during Wheat Caryopsis Development under Drought Stress

**DOI:** 10.3389/fpls.2017.01707

**Published:** 2017-10-04

**Authors:** Xin-yu Chen, Yang Yang, Li-ping Ran, Zhao-di Dong, Er-jin Zhang, Xu-run Yu, Fei Xiong

**Affiliations:** Jiangsu Key Laboratory of Crop Genetics and Physiology, Co-Innovation Center for Modern Production Technology of Grain Crops, Joint International Research Laboratory of Agriculture and Agri-Product Safety, Yangzhou University, Yangzhou, China

**Keywords:** wheat, drought stress, microRNA, storage protein, protein body

## Abstract

Drought stress is a significant abiotic stress factor that affects wheat yield and quality. MicroRNA (miRNA) plays an important role in regulating caryopsis development in response to drought stress. However, little is known about the expression characteristics of miRNAs and how they regulate protein accumulation in wheat caryopsis under drought stress. To address this, two small RNA libraries of wheat caryopsis under control and drought stress conditions were constructed and sequenced. A total of 125 miRNAs were identified in the two samples, of which 110 were known and 15 were novel. A total of 1,981 miRNA target genes were predicted and functional annotations were obtained from various databases for 1,641 of them. Four miRNAs were identified as differential expression under drought stress, and the expression patterns of three of them were consistent with results obtained by reverse transcription polymerase chain reaction (RT-PCR) and reverse transcription quantitative polymerase chain reaction (RT-qPCR). Moreover, three miRNA-target pairs showed negative regulation tendency, as revealed by RT-qPCR. Functional enrichment and pathway analysis revealed that four pathways might be involved in storage protein biosynthesis. Furthermore, drought stress significantly increased the accumulation of protein bodies and protein content in wheat endosperm. In summary, our findings suggest that drought stress may enhance storage protein by regulating the expression of miRNAs and their target genes.

## Introduction

Drought stress is a major abiotic stress factor that poses a significant challenge to global crop production. Long term or periodic droughts greatly affect crop growth and yield formation. Therefore, investigating drought resistance of crops is critical for achieving long-term productivity gains (Lawlor, 2013). Wheat is an important cereal crop that is widely cultivated around the world and is sensitive to drought stress during vegetative and reproductive growth (Liu et al., 2005). In response to drought stress, wheat exhibits systematic adaptability in morphological structure, physiology, biochemistry, and gene regulation (Chaves et al., 2003). When subjected to drought stress, expression of relevant stress-resistant genes is induced and regulated by molecule-mediated sensory and conduction signaling systems. These include effector and regulatory molecules that are directly involved in biochemical responses and expression products of non-encoding genes in wheat (Shinozaki and Yamaguchi-Shinozaki, 2007).

MicroRNA (miRNA), an endogenous non-coding single-stranded RNA, is highly conserved and widely present in eukaryotes. Mature miRNAs, which are usually 21–24 nucleotides in length, are a kind of important regulatory factor of gene expression in eukaryotes (Ambros, 2004). Transcription of miRNA-encoding genes is catalyzed by RNA polymerase II, and the transcripts are spliced into mature miRNA via dicer-like enzymes and several protein complexes (Bartel, 2004). Based on complementarities between miRNA and target genes, miRNAs regulate target gene expression via two mechanisms, namely, cleavage-induced targeted mRNA degradation and translational repression (Mallory and Bouché, 2008). In plants, most miRNAs display perfect or near perfect complementarity with their mRNA targets. Thus, plant miRNAs regulate the expression of target genes primarily through specific mRNA cleavage (Rhoades et al., 2002; Chen, 2005).

miRNA plays an essential role in plant development and stress adaptation. By performing high-throughput sequencing of all small RNAs in samples, miRNA expression profiles can be obtained at the whole genome level without any sequence information. This can then be used for discovery of novel miRNAs, prediction of target genes, and differential expression analysis, which may greatly promote the discovery and functional study of plant miRNAs (Buermans et al., 2010). Recent studies have shown that miRNAs are involved in regulating responses to various biotic and abiotic stresses in plants (Covarrubias and Reyes, 2009; Jagadeeswaran et al., 2009). In rice, miR169g expression is significantly up-regulated under drought stress, whereas miR393 only exhibits transient expression (Zhao et al., 2007). High-throughput sequencing was used to study the small RNAs in potatoes under drought stress. Numerous conserved and specific miRNAs were identified and a negative correlation between drought-related miRNAs and target genes was found (Zhang et al., 2014). It is widely believed that stress conditions change the expression of miRNAs, which in turn leads to changes in expression of miRNA targets (Reyes and Chua, 2007). Thus, functional analysis of targets can be helpful for understanding the mechanism underlying the regulatory role of miRNA in stress responses. Song et al. (2012) identified the target of miR394 which encoded an F-box protein (At1g27340) involved in the regulation of leaf curling-related morphology in Arabidopsis. Additionally, miRNA can affect plant growth under stress conditions by regulating signaling pathways. MiR393 was found to target transport inhibitor response 1 (TIR1), which is known as an auxin receptor and positive regulator of auxin signaling (Windels and Vazquez, 2011). To validate these target genes of miRNA, degradome sequencing can be used (Zhou M. et al., 2010). Li et al. (2015) investigated development-related miRNAs and their validated targets during wheat grain development by small RNA sequencing combined with degradome sequencing.

Wheat caryopsis is an important organ for nutrient storage, and its development determines the yield and quality of wheat. Drea et al. (2005) carried out a systematic analysis of gene expression during caryopsis development and revealed novel distinct spatial expression patterns that either indicated the ontogeny of the developing caryopsis or reflected specialized cellular function. Protein, which is one of the main storage substances in wheat endosperm, accounts for 10–15% of the dry weight of mature wheat seeds (Tasleem-Tahir et al., 2012). In wheat, as in other cereal crops, storage proteins, including gliadin, glutenin, and globulin, accumulate in an endosperm organelle with the development of caryopsis, protein body (PB), which may be formed in the rough endoplasmic reticulum or protein storage vacuoles (Chen et al., 2014). Protein content and PB amount directly affect the processing and nutritional quality of wheat flour (Kindred et al., 2008). The biosynthesis of storage protein is generally considered to be regulated at the transcriptional level (Verdier and Thompson, 2008). Previous studies have indicated that some genes are not directly involved in storage protein gene expression but may influence storage protein biosynthesis, such as those encoding glutamate synthetase and glutamine synthase (Habash et al., 2009). However, the expression characteristics and regulation mechanism of miRNAs during wheat caryopsis development, particularly the protein biosynthesis and PB development under drought stress, remain to be unknown. To investigate this issue, high-throughput sequencing of miRNA was conducted, and the differentially expressed miRNAs in wheat caryopsis under drought stress were identified. In addition, the miRNA target genes were predicted and functional analysis of target genes was performed to investigate the possible mechanism underlying miRNA regulation of storage protein biosynthesis in wheat endosperm when subjected to drought stress. Our study may inform future studies on mechanisms underlying wheat drought resistance and may provide a theoretical basis for genetic breeding of drought-tolerant wheat cultivars.

## Materials and methods

### Plant materials and treatment

**Figure 1:**
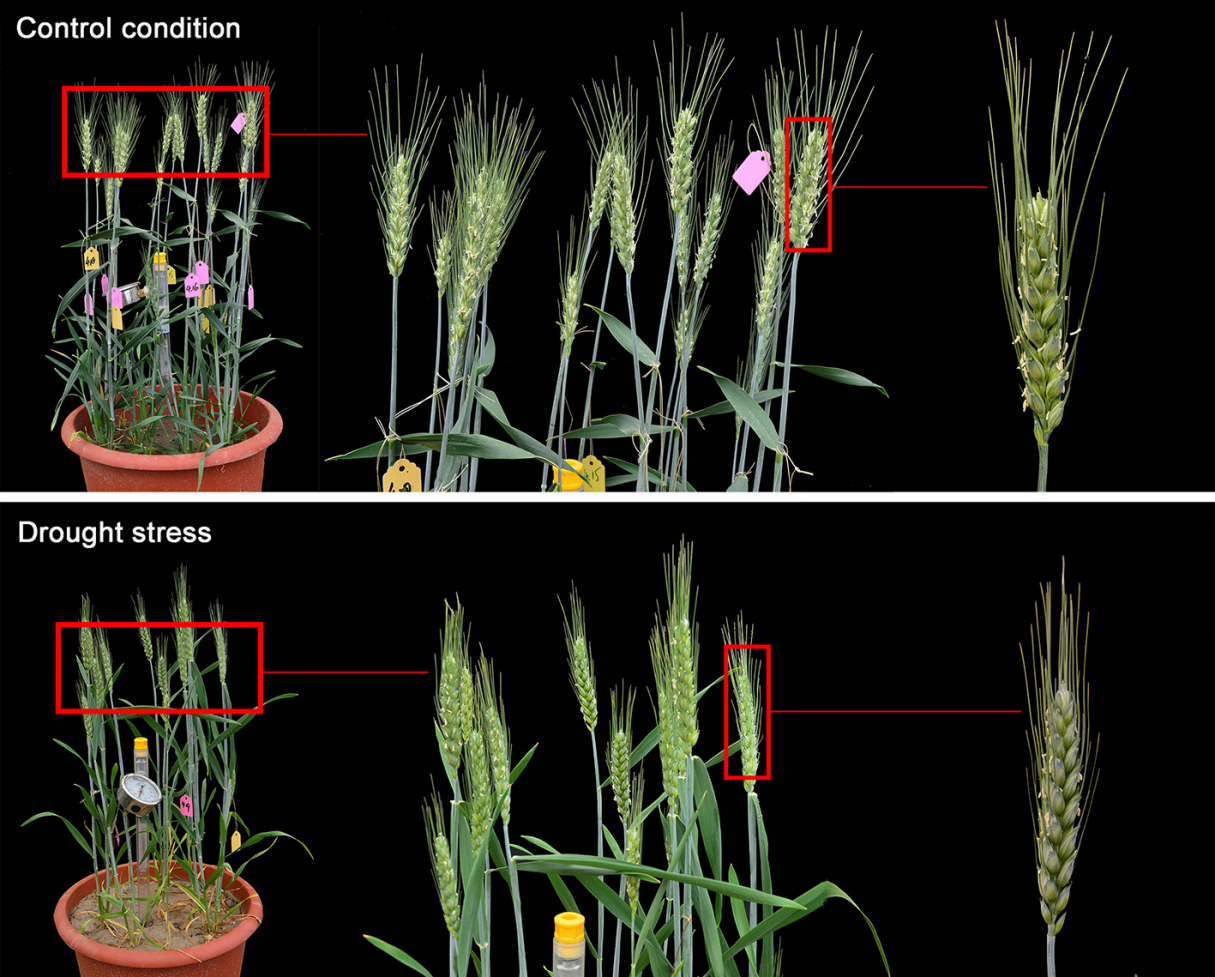
Wheat plant under control and drought stress conditions. Wheat was planted in plastic pots maintaining soil water potential at −20 and −60 kPa, which corresponded to control and drought stress conditions, respectively. Under control condition, wheat plant grew well while wheat leaves grew yellow under drought stress. Meanwhile, the wheat ears under drought stress were smaller and the grains were not as full as those under control.

The wheat cultivar selected in this study was Yangmai 16 provided by the Agricultural College of Yangzhou University, Jiangsu Province, China. Seeds were sown in plastic pots (20 seeds per pot) that were placed in rainproof shelters in the experimental field of Key Laboratory of Crop Genetics and Physiology in Yangzhou University from 2015 to 2016. The soil was sandy loam and contained organic substances (2.45%), nitrogen (106 mg/kg), phosphorus (33.8 mg/kg), and available potassium (66.4 mg/kg). Seedlings were thinned to eight plants per pot 2 weeks after sowing. A minupressure soil hygrometer (SP-11, Institute of Soil Science in Nanjing, China) was used to detect the water potential at 15 cm in the soil. Watering was strictly controlled from plant re-greening (the stage before erecting and after wintering) to the caryopsis mature stage, maintaining water potential at −20 and −60 kPa. These values corresponded to the optimum level of normal water supply condition and drought stress, respectively (Figure 1). All other steps for management of the control and treatment groups throughout the growth cycle were the same. Each treatment group consisted of 30 pots. During wheat flowering, two individual florets at the base of the central ears were marked with a marker pen to label the anthesis date.

### RNA extraction and sequencing

Wheat caryopses under control and drought stress were collected 15 days after anthesis (DAA) and frozen immediately in liquid nitrogen. The collected samples were made up of caryopses from the middle of six different wheat ears and each two ears were from three different plastic pots. Frozen tissues were pulverized in liquid nitrogen using a mortar and pestle. Total RNAs were extracted using Trizol Reagent (Shanghai Sangon Biotech Co. Ltd., China) following the manufacturer's instructions. The purity, concentration, and integrity of RNA samples were assayed using Nanodrop, Qubit2.0, and Agilent 2,100 bioanalyzer, respectively. Two small RNA libraries were constructed using the small RNA Sample Pre Kit. Briefly, the extracted total RNA was ligated to 5′ and 3′ adapters using T4 RNA Ligase 1 and T4 RNA Ligase 2, respectively. Subsequently, the cDNA was synthesized by reverse transcription and amplified using polymerase chain reaction (PCR). Then the products were purified via PAGE to attain small RNA libraries. Finally, the quality control of libraries was performed using Qubit 2.0, Agilent 2,100 bioanalyzer, and quantitative PCR. The resulting RNA was sequenced by Nanjing Genepioneer Biotechnologies using the Illumina HiSeq2500 high-throughput sequencing platform.

### Basic analysis of sequencing data

The raw image data files from sequencing were converted to raw data via base calling, and clean reads were obtained by filtering out the 5′ terminal contamination or low quality sequences. The effective reads were eventually obtained after quality control of the clean reads. The effective reads were aligned to Rfam and Repbase databases using Bowtie (Langmead, 2010) software. The small RNA was annotated according to the priority order of rRNA > tRNA > snRNA > snoRNA > repeat. The unclassified ncRNA tags were aligned with the reference genome using miRDeep2 software (Friedländer et al., 2012) to obtain the position information on the reference genome. Then, sequence length distribution as well as common and specific sequence analysis were conducted.

### miRNA analysis

Sequences mapped to reference genome were used for known and novel miRNA identification using miRDeep2 software. The effective reads mapped to reference genomes were aligned against precursor and mature sequences of known wheat miRNA in miRBase. If precursor sequence, mature sequence, and hairpin structure were consistent, the effective reads were regarded as known miRNAs. Novel miRNA prediction was done using miRDeep2 software, which can identify novel miRNA by predicting hairpin structure, analyzing Dicer enzyme splice sites, and calculating free energy. Moreover, length distribution statistics and base preference analysis of identified miRNAs were performed.

### Differential expression analysis of miRNA

To identify the differentially expressed miRNAs, expression levels of miRNAs were compared between the control and drought stress samples. Based on the sequencing data, miRNA expressions in the two samples were counted and normalized to obtain transcript per million using a normalization formula described previously (Zhou L. et al., 2010). Then, the fold change and *p*-values were calculated from the normalized expressions. EdgeR was used to conduct the differential expression analysis, which ran a web tool IDEG6 to detect differentially expressed miRNAs (Romualdi et al., 2003). The differentially expressed miRNAs were screened at a standard of |log2(FC)| ≥ 1 and *p*-value < 0.05.

### Reverse transcription PCR (RT-PCR) of differentially expressed miRNA

Total RNA was extracted from wheat caryopses under control and drought conditions using Trizol reagent according to the manufacturer's instructions. RNA was reverse transcribed to cDNA using One-Step miRNA RT Kit (SinoGene, China). The reverse transcription reaction mixture contained 12 μL RNA template mix, 1 μL Oligo-dT adapter primer, 4 μL 5 × reaction buffer, 1 μL M-MLV RTase and 2 μL 10 mM of dNTP. The reverse transcription reaction was performed at 37°C for 60 min followed by 85°C for 10 min. RT-PCR was performed for 4 miRNAs using cDNA as template and *U6* served as internal control to normalize the cDNA concentrations. PCR amplification was performed in a 25 μL reaction volume containing 0.5 μL Taq enzyme, 2.5 μL cDNA template, 3 μL 10 × buffer (including MgCl_2_), 1 μL dNTP, 1 μL of each primer at the concentration of 20 mM, and 11 μL nuclease-free ddH_2_O. The condition of PRC was set as follows: 95°C for 3 min, followed by 25–35 cycles of 94°C for 30 s, 52°C for 30 s, and 68°C for 30 s, depending upon the individual miRNA. Final extension was performed at 68°C for 7 min. Amplified products were separated on 1.5% agarose gel and the marker used was NormalRun™ prestained 250 bp-I DNA ladder (Generay, China).

### Prediction and functional annotation of miRNA target genes

The target genes of miRNA were predicted using psRNATarget (Enright et al., 2003) according to sequence alignment between identified miRNAs and wheat genomes. Non-redundant protein sequences, Swiss-Prot, gene ontology (GO), Cluster of Orthologous Groups of proteins, Kyoto Encyclopedia of Genes and Genomes (KEGG), Eukaryotic Ortholog Groups, and Pfam database were used to annotate the function of target genes using BLAST software. GO enrichment (Consortium et al., 2000) and KEGG pathway analysis (Kanehisa et al., 2004) were undertaken on target genes of differentially expressed miRNAs.

### Microstructure observation of wheat caryopsis

Wheat caryopses at 15 DAA were acquired and cut transversely into 2 mm slices from the middle using a razor blade. The caryopsis slices were soaked in 2.5% glutaraldehyde fixative [25% glutaraldehyde diluted 10 times at pH 7.2 phosphate-buffered solution (PBS)] at 4°C for 48 h immediately. The fixed samples were subsequently rinsed thrice with PBS and dehydrated in a graded ethanol series [20, 40, 60, 80, 90, 95, and 100% (thrice)], followed by propylene oxide replacement. Afterward, the samples were infiltrated and embedded in low-viscosity Spurr's resin and polymerized at 70°C for 12 h. The samples were cut into 1 μm slices using an ultramicrotome (Ultracut R, Leica, Germany) and pasted onto glass slides. Then, the slices were stained with 0.5% methyl violet, rinsed, dried, and observed under a light microscope (DMLS, Leica, Germany). Photographs were captured using a camera (Truechrome II, Truechrome, China) attached to the light microscope. Each treatment selected three caryopses as repeats, each of which was from different wheat spikes.

### Protein content determination

Wheat caryopses were collected at 15 and 45 DAA (mature stage) and dried at 42°C to a constant weight. The samples were then completely ground using a mortar and the powder was filtered through a 100 mesh wire screen. Up to 200 mg of the sample was mixed with the catalyst (CuSO_4_ + K_2_SO_4_). The nitrogen content (%) was determined on an automatic Kjeldahl apparatus (FOSS KjeltecTM8400, Analyzer, Unit, Hoganas) after digesting the concentrated sulfuric acid. Sample protein content (%) was calculated by multiplying the coefficient 5.7. Three individual replicates were used for each sample.

### Reverse transcription quantitative PCR (RT-QPCR) analysis of differentially expressed miRNAs and target genes

Total RNA was extracted from both control and drought-treated samples using Trizol reagent according to the manufacturer's instructions. For miRNA, 5 μg of total RNA was polyadenylated with a poly (A) adapter and reverse-transcribed into cDNA using One-Step miRNA RT Kit (SinoGene, China). The sequence complementary to the poly(A) adapter was used as the primer. Reverse transcription reaction was performed in 20 μL reaction mixture (RNA Template Mix 12 μL, Oligo-dT Adapter Primer 1 μL, 5 × Reaction Buffer 4 μL, M-MLV RTase 1 μL, 10 mM of dNTP 2 μL) at 37°C for 60 min followed by 85°C for 10 min. For target gene, 5 μg of total RNA was reverse-transcribed into cDNA using PrimeScript™ RT reagent Kit (TaKaRa, Japan). Reverse transcription reaction was performed in a 20 μL reaction mixture containing total RNA mix, 4 μL 5 × PrimeScript buffer (including dNTP mixture and Mg^2+^), 1 μL PrimeScript RT enzyme mix I, 1 μL oligo dT primer, 1 μL random 6 mers, and RNase-free ddH_2_O up to 20 μL. The condition for reverse transcription reaction was set at 37°C for 15 min followed by 85°C for 1 min.

RT-qPCR of miRNAs and their target genes was performed on an ABI 7,500 Real-Time PCR system using SYBR Premix Ex Taq™ and SYBR Green (TaKaRa, Japan) following the manufacturer's instructions. PCR reactions of miRNAs were performed at 95°C for 10 min, followed by 40 cycles of 95°C for 20 s, 60°C for 30 s, and a cycle of dissociation at 95°C for 15 s, 60°C for 30 s, 95°C for 15 s. PCR reactions of target genes were performed as follows: 95°C 1 min; 40 cycles of 95°C 10 s, 60°C 30 s, and 72°C 30 s. The primers used are listed in Table 1. *U6* (a kind of snRNA that forms ribonucleoprotein) and wheat unigene *Ta54825* (Actin) were used as internal control to analyze the relative expression level of miRNAs and target genes, respectively. Calculation of relative expression levels of genes was done by the ΔΔ^Ct^ method (Pfaffl, 2001). The expression levels of genes are presented as means calculated from three replicates.

**Table 1:** Primers for internal control genes, differentially expressed miRNAs and corresponding target genes used in RT-qPCR validation.

**Type**	**Gene/miRNA**	**Forward primer 5′ → 3′**	**Reverse primer 5′ → 3′**
Target gene	*Ta54825*	TGACCGTATGAGCAAGGAG	CCAGACAACTCGCAACTTAG
	*TC436629*	CGACTGCTTCTCCTACCT	GATGCCGTTGAGGACCT
	*TC369499*	GTCGCTCCATTGCCTACTT	GAACCTGGGCTCATTGTCC
	*TC371915*	GGCGTGCTTCTGGGTAT	GCTTCAAATGTGGTGGG
	*DR733425*	GCTCCACCTACATTCTCG	CTCTGCTGCTTTCTCAA
miRNA	*U6*	cttcggggacatccgataaaattg	–
	tae-nsmR10	atatAGGCGCCCTGGGGGGCCGAACGGC	–
	tae-csmR5082-1	tatataCGCGAtGAtGGCCGCGCGGGCtCAC	–
	tae-nsmR5/6	AAACCCGGACtGtGtCGtAtGtGC	–
	tae-miR9654a-3p	gcttCtGAAAGGCttGAAGCGAAt	–

*Lowercase, RNA; uppercase, DNA. The reverse primers used for RT-qPCR of miRNAs are provided by the kit. Sequences are not listed in this study to preserve product confidentiality*.

### Statistical analysis

Data analyses were conducted using Excel 2016 (Microsoft, USA). *T*-test was used to compare the means at a significance level of *p* < 0.05. Origin 8.0 (OriginLab, USA) and Photoshop CS5 (Adobe, USA) were used to illustrate the figures.

## Results

### Sequence analysis of small RNAs

After sequencing, 15,536,948 and 10,340,923 effective reads were obtained from wheat caryopses under control and drought stress, respectively. Most of effective reads were annotated into different classes and mapped to the reference genomes (Table 2). As a result, the total tags and unique tags mapped to the reference genomes accounted for the largest proportion, followed by the percentage of total tags and unique tags annotated as rRNA. The rRNA amount can be used as a quality control standard for samples, and the rRNA proportion in plant RNA samples is generally <60%. Up to 38.22% of the total tags and 8.07% of the unique tags were annotated as rRNA in the control group. Moreover, the proportion of total tags and unique tags annotated as rRNA in the drought-treated group were 42.62 and 9.71%, respectively. This indicated that our sequencing data were of good quality. Up to 7,272,595 (46.81%) total tags and 3,664,651 (75.67%) unique tags in the control group were mapped to the reference genomes. Meanwhile, in the treatment group, the total tags and unique tags mapped to the reference genome were 4,574,247 (44.23%) and 2,549,296 (74.43%), respectively. These effective reads mapped to the reference genomes were used for subsequent miRNA identification.

**Table 2:** Statistics of small RNA classification annotation.

**Type**	**Control**				**Drought stress**			
	**Total tags**	**Percent %**	**Unique tags**	**Percent %**	**Total tags**	**Percent %**	**Unique tags**	**Percent %**
rRNA	5938872	38.22	391002	8.07	4407033	42.62	332457	9.71
tRNA	312093	2.01	31109	0.64	184818	1.79	23654	0.69
snRNA	23444	0.15	8931	0.18	14992	0.14	6798	0.20
snoRNA	63273	0.41	14541	0.30	38836	0.38	10872	0.32
Repeat	813246	5.23	315419	6.51	554829	5.37	231042	6.75
Mapped	7272595	46.81	3664651	75.67	4574247	44.23	2549296	74.43
Unmapped	1113425	7.17	417220	8.62	566168	5.48	270962	7.91
Total	15536948	100	4842873	100	10340923	100	3425081	100

**Figure 2:**
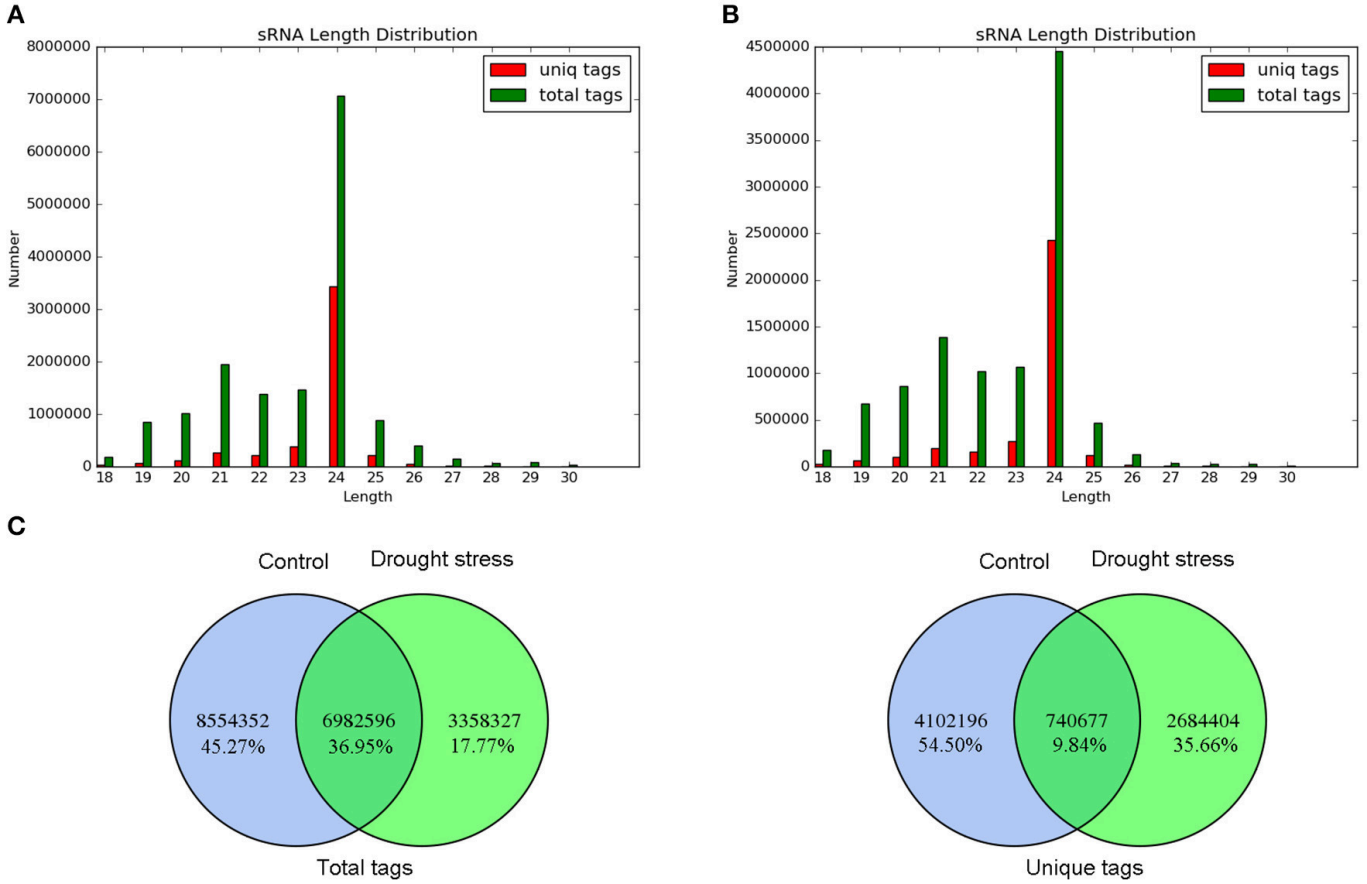
Sequence analysis of small RNAs. **(A)** Length distribution of small RNAs under control. **(B)** Length distribution of small RNAs under drought stress. The distributions of total tags and unique tags are shown as numbers. **(C)** Venn diagram of common and specific sequence statistics between control and drought stress. Total tags and unique tags are shown in the left and right panels, respectively.

The length distribution of small RNAs and common and specific sequence analysis of total tags and unique tags between control and drought stress are shown in Figure 2. Under control and drought conditions, the length distributions of the total tags and unique tags were basically similar but uneven. The number of total tags and unique tags with 24 nt in length was the largest, and the rest were mainly distributed between 19 and 23 nt (Figures 2A,B). These results are similar to the length distribution of small RNAs in plants. Furthermore, 6,982,596 (36.95%) total tags and 740,677 (9.84%) unique tags were the common sequences between the two samples (Figure 2C). This indicated good consistency of the samples in sequencing, and that the sequencing results were indeed of good representativeness.

### Identification, length distribution statistics, and base preference analysis of miRNAs

**Figure 3:**
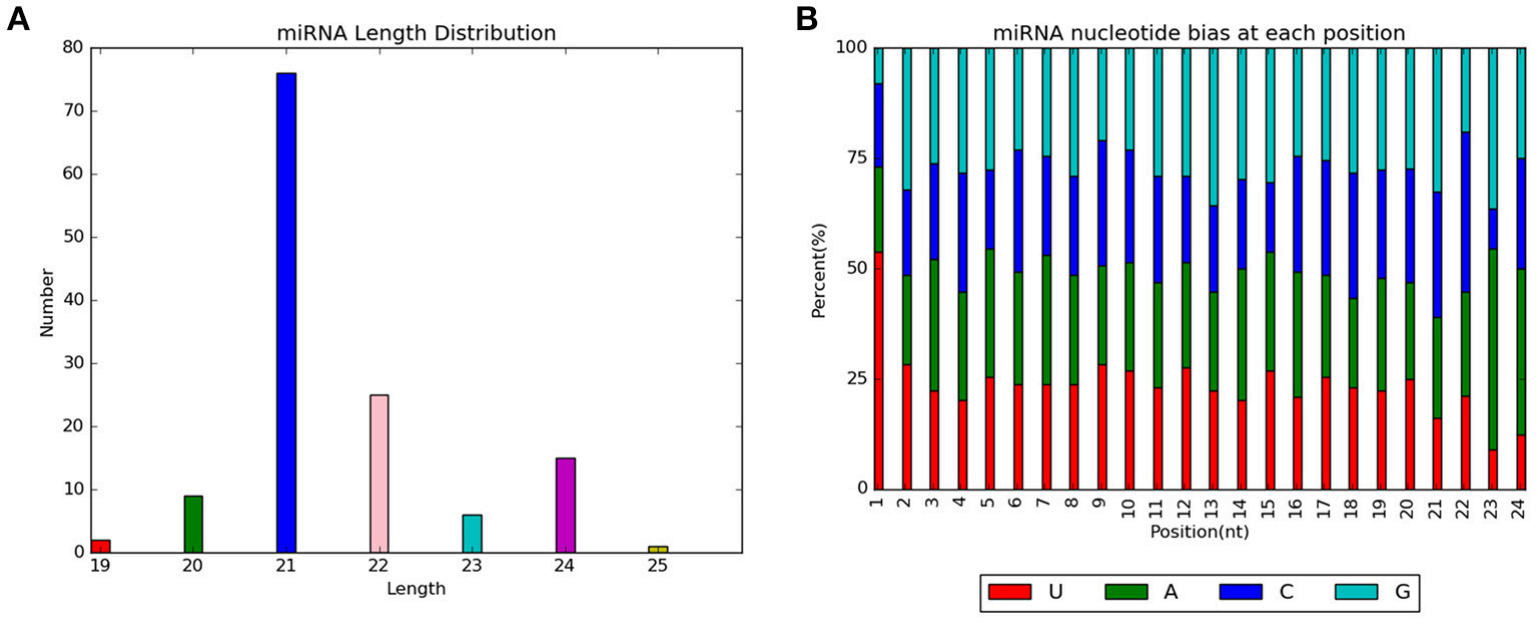
Analysis of all identified miRNAs from control and drought stress samples. **(A)** Length distribution of all miRNAs. **(B)** Nucleotide biases at each position for all miRNAs.

By aligning with known miRNAs in miRBase and predicting novel miRNAs, 125 miRNAs were identified in the control and drought stress groups. This included 110 known miRNAs and 15 novel putative miRNAs [miRNA specific sequence information is shown in Supplementary Material 1 (Table)]. Specific expression of tae-miR399 was found in the control sample, whereas tae-miR5085, tae-miR5200, and tae-miR6197-5p were specifically expressed in the drought sample. Length distribution of all identified miRNAs showed that the majority of miRNA lengths were in the range of 20–24 nt (Figure 3A). Considering that dicer enzymes specifically recognize and cleave precursor miRNAs, the first base site of mature miRNA displayed strong preference to U. Moreover, the other sites displayed a certain base bias, which can be used to evaluate the sequencing accuracy. The base distribution analysis of miRNA at each site showed that the content of C was low, and the contents of G, U, and A were similar (Figure 3B).

**Figure 4:**
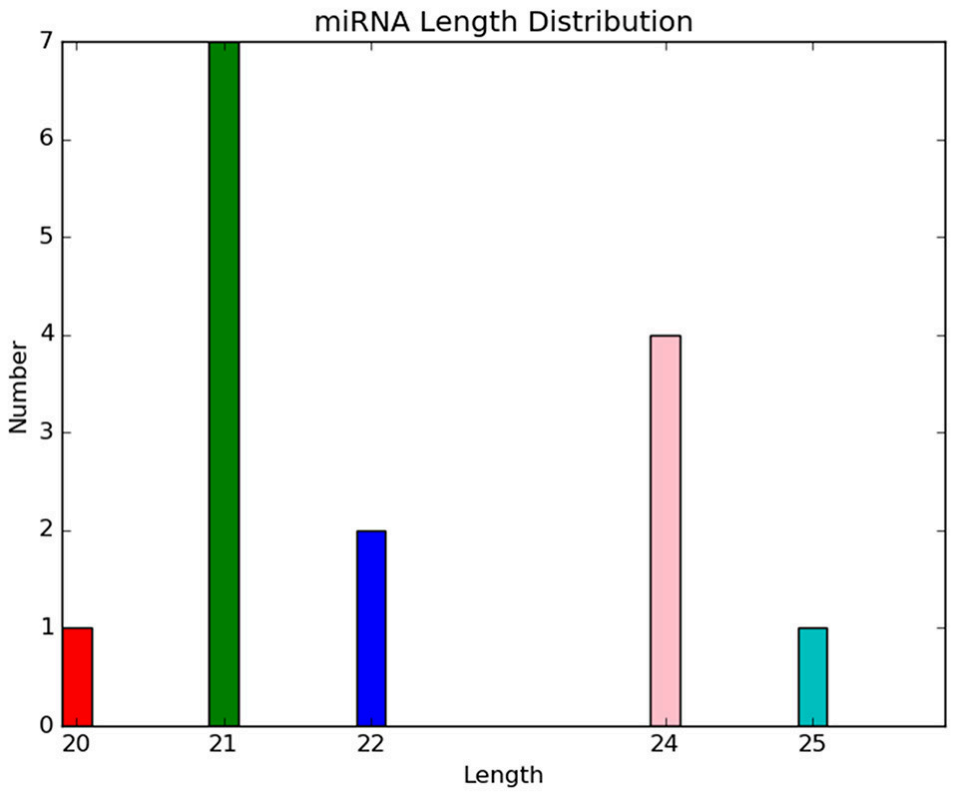
Length distribution of novel putative miRNAs in control and drought stress samples.

Five novel putative miRNAs were classified as conservative miRNAs, and 10 were unconservative miRNAs. Their specific sequence information is shown in Table 3. Length distribution analysis showed that the novel miRNAs with a length of 20 nt were the most followed by 24 nt miRNAs (Figure 4). The novel putative miRNA precursor structure was predicted using the miRDeep2 software. Detailed information is shown in Supplementary Material 2 (Figure).

**Table 3:** Sequence information of putative novel miRNA.

**Conservative miRNA**		**Unconservative miRNA**	
**miRNA name**	**Mature miRNA sequence**	**miRNA name**	**Mature miRNA sequence**
tae-csmR156-1	UGACAGAAGAGAGUGAGCAC	tae-nsmR1	UCGGCUACUUCCUUUCCCUUGC
tae-csmR5082-1	CGCGAUGAUGGCCGCGCGGGCUCAC	tae-nsmR2	UCACUGUUUGGAAAGGUAGGAC
tae-csmR7743-1	AUUGAACUAAGGAGGGGUGGA	tae-nsmR3	CGCGGCUCCGUCGACUGGUGC
tae-csmR172-1	GCAGCACCACCAAGAUUCACA	tae-nsmR4	GUCUCUGCCAAUUCUUCGUGU
tae-csmR2275-1	UUUGGUUUGAAGGGAGCUCUG	tae-nsmR5	AAACCCGGACUGUGUCGUAUGUGC
		tae-nsmR6	AAACCCGGACUGUGUCGUAUGUGC
		tae-nsmR7	AGCCCGCGGCAUGGCGUCGCA
		tae-nsmR8	UUAGGAACAUGUGUGCUCUCUUGU
		tae-nsmR9	CAUCCCCUUCGCCGGCUGCGC
		tae-nsmR10	AGGCGCCCUGGGGGGCCGAACGGC

### Differential expression analysis of miRNA

**Figure 5:**
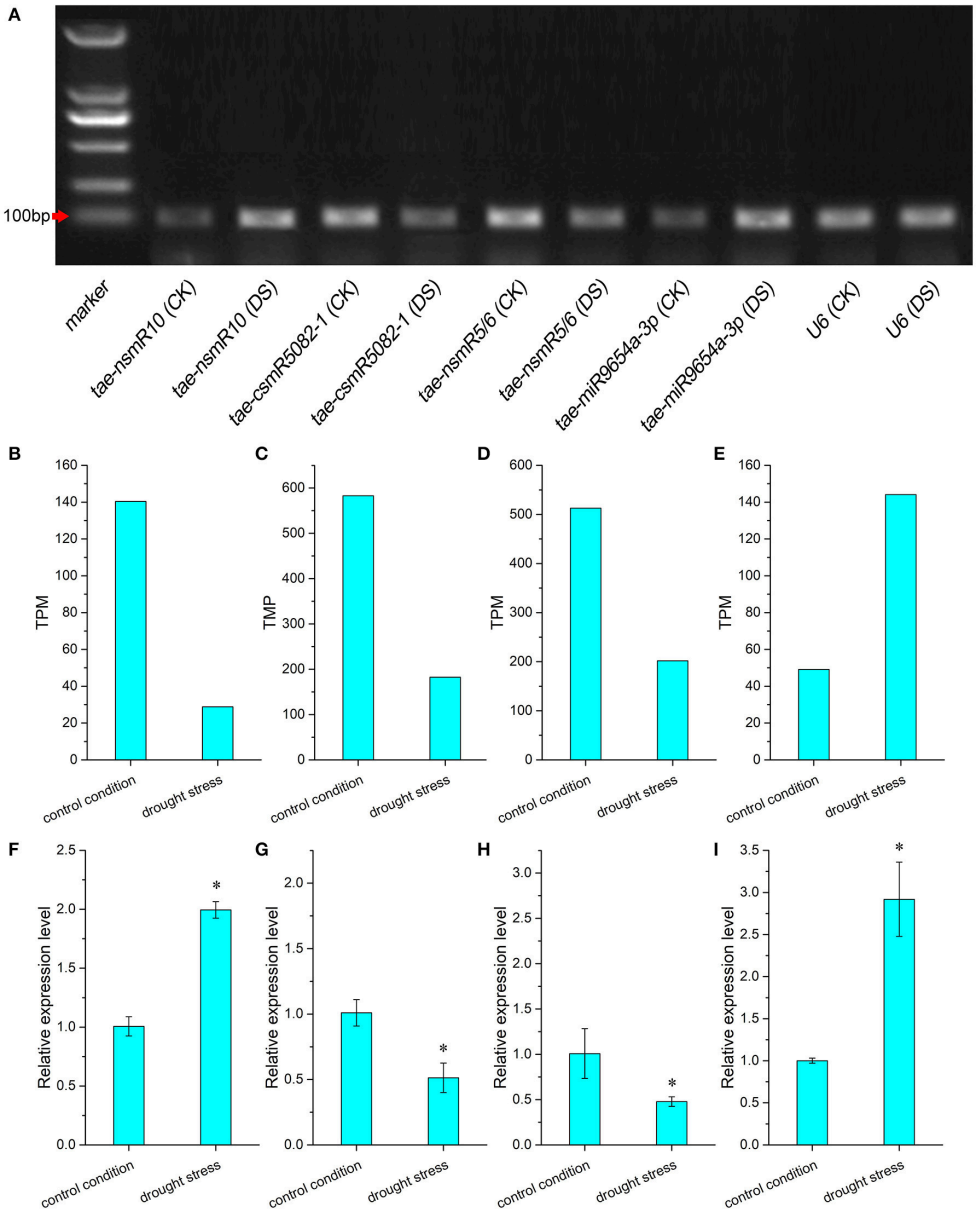
Differential expression analysis of miRNA in caryopses under drought stress. **(A)** Expression profile of differentially expressed miRNA by RT-PCR. CK indicates control condition and DS indicates drought stress. **(B,F)** TPM value and relative expression level of tae-nsmR10; **(C,G)** TPM value and relative expression level of tae-csmR5082-1; **(D,H)** TPM value and relative expression level of tae-nsmR5/6; **(E,I)** TPM value and relative expression level of tae-miR9654a-3p. Each miRNA expression value was normalized against the internal control gene *U6*. Relative expression levels were presented as mean ± SE, and means were calculated from three replicates. Asterisks between the control and drought stress indicate significant difference at *p* < 0.05 as determined by *t*-test.

Expression analysis of miRNA under drought stress is shown in Figure 5. Differences in miRNA expression profiles were investigated by RT-PCR (Figure 5A). The transcript signals of miRNA tae-nsmR10 and tae-miR9654a-3p in caryopses under drought stress were higher than those in control caryopses. However, transcript accumulations of miRNA tae-csmR5082-1 and tae-nsmR5/6 were decreased under drought stress. The TPM value and relative expression levels of differentially expressed miRNAs in control and drought samples were calculated. Based on high-throughput sequencing data and bioinformatic analysis, four miRNAs with significantly differential expression were singled out, namely, tae-nsmR10, tae-csmR5082-1, tae-nsmR5/tae-nsmR6 (Tae-nsmR5 and tae-nsmR6 were considered to be the same miRNA because they had the same sequence) and tae-miR9654a-3p. The expression of known miRNA tae-miR9654a-3p was up-regulated, whereas the other three novel miRNAs were down-regulated after drought stress treatment (Figures 5B–E). To further validate the expression profile of differentially expressed miRNA under drought stress, the relative expression levels of the above four miRNAs were analyzed by RT-qPCR. The relative expression level of tae-nsmR10 and tae-miR9654a-3p exhibited the up-regulated pattern while that of tae-csmR5082-1 and tae-nsmR5/tae-nsmR6 exhibited the down-regulated pattern under drought stress (Figures 5F–I). In addition, the expression trend of three miRNAs (tae-csmR5082-1, tae-nsmR5/tae-nsmR6 and tae-miR9654a-3p) showed consistent results between the TPM value and relative expression level determined by high-throughput sequencing and RT-qPCR, respectively (Figures 5C,G,D,H,E,I). However, for tae-nsmR10, the TPM value was inconsistent with the relative expression level (Figures 5B,F).

### Prediction and functional analysis of differentially expressed miRNA target genes

Target genes of all identified miRNAs were predicted using psRNATarget software and compared with the relevant database for functional annotation. For all the identified miRNAs, 1,981 target genes were predicted. Of these, 1,641 target genes were annotated. Interestingly, each miRNA can have different targets, and different miRNAs can have the same target gene. For differentially expressed miRNAs, 28 predicted target genes were annotated with function and specific information as shown in Table 4.

**Table 4:** Prediction and functional annotation of differentially expressed miRNA targets.

**miRNA ID**	**Targets ID**	**Annotation**
tae-csmR5082-1	TC390380	Cationic peroxidase SPC4
	CA637513	Arabinogalactan peptide
	CA644040	Arabinogalactan peptide
	CA634690	Arabinogalactan peptide
	TC436117	Arabinogalactan peptide
	CA645741	IAA-amino acid hydrolase
	TC389427	Predicted protein
	TC438668	Hypothetical protein
	TC449449	Hypothetical protein
	CA610228	Peroxidase
	CA663463	RUBISCO activase beta
	TC395269	beta-1,3-galactosyltransferase
	TC432653	DEAD-box ATP-dependent RNA helicase
	**TC369499**	1-aminocyclopropane-1-carboxylate oxidase
tae-nsmR5, tae-nsmR6	TC435511	3-mercaptopyruvate sulfurtransferase
	**TC371915**	3-mercaptopyruvate sulfurtransferase
tae-nsmR10	**TC436629**	Proline-rich receptor
	CA678419	Peroxiredoxin-2C
	DR735664	SNF1-related protein kinase catalytic subunit
	TC458111	Hypothetical protein
	CA646062	Peroxiredoxin-2C
	CJ793000	Trehalose-phosphate phosphatase
tae-miR9654a-3p	TC422325	Sulfated surface glycoprotein
	DR735126	Shikimate dehydrogenase
	**DR733425**	Vesicle-associated protein
	BJ258028	Predicted protein
	CJ698196	Predicted protein
	TC426820	Heterogeneous nuclear ribonucleoprotein

**Figure 6:**
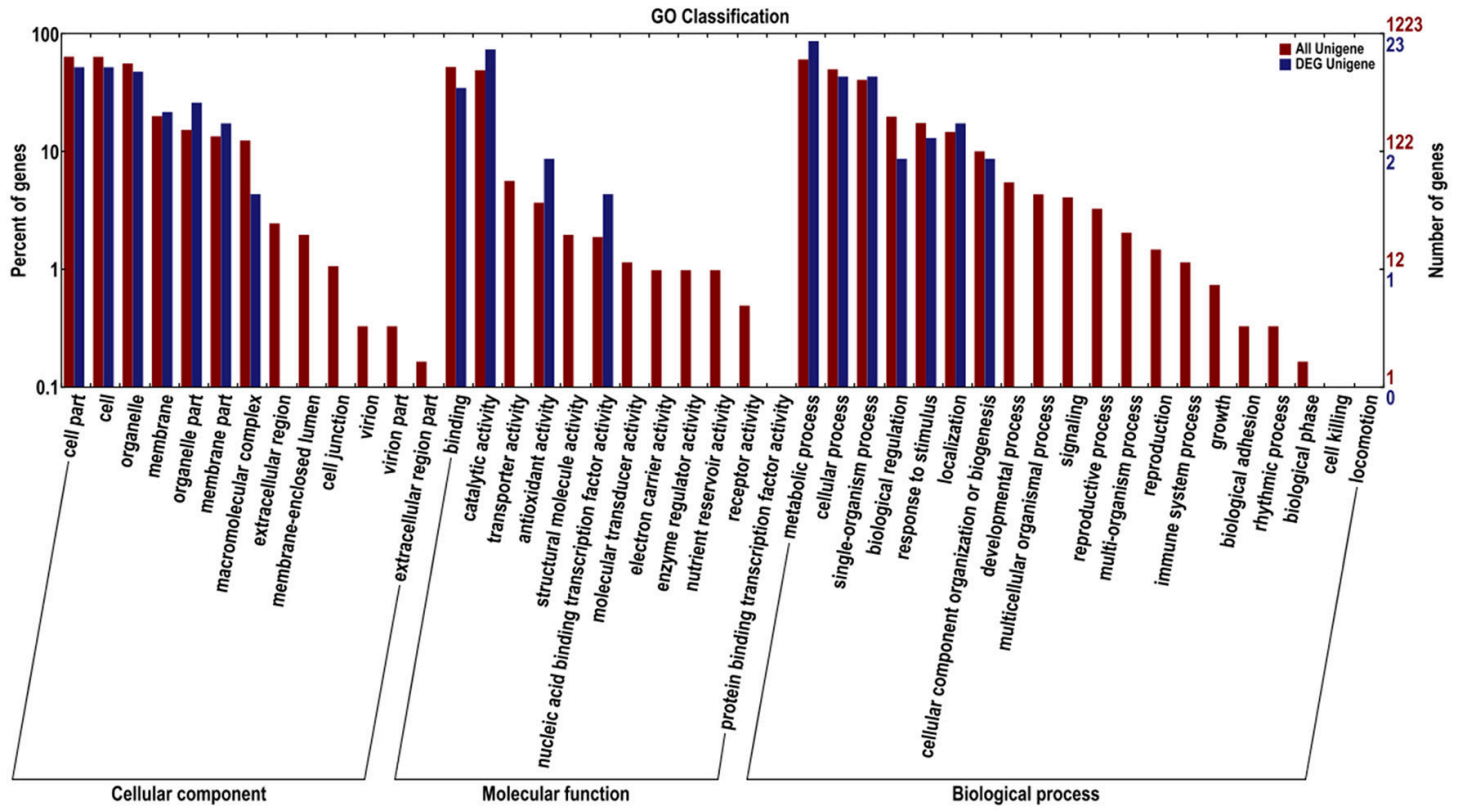
GO enrichment functional analysis of miRNA targets. Red represents target genes for all miRNAs, and blue represents target genes for differentially expressed miRNAs.

Functional annotation based on GO and KEGG databases revealed that miRNA targets had various functions, involving biological processes, molecular genetics and other aspects. Moreover, GO enrichment analysis of all identified miRNAs and differentially expressed miRNA targets was performed and the results are shown in Figure 6. According to GO functional annotation, the target genes of miRNA were classified into three categories, namely, biological process, cellular component, and molecular function. For the cellular component category, numerous target genes for all identified and differentially expressed miRNAs fell under cell part, cell, organelle, membrane, organelle part, membrane component, and macromolecular complex subcategories. Moreover, the extracellular region comprised the smallest proportion of all miRNA targets. In the molecular functional category, the two most abundant subcategories for all miRNAs were binding and catalytic activity. The target genes of differentially expressed miRNAs were classified under binding, catalytic activity, antioxidant activity, and nucleic acid binding transcription factor activity. Under the biological process category, majority of all miRNA targets and all the differentially expressed miRNA targets were enriched under the following secondary function entries: metabolic processes, cellular process, single-organism process, biological regulation, response to stimulus, localization, and cellular component organization or biogenesis. Moreover, the enrichment trends of all miRNA targets and differentially expressed miRNA targets in GO entries were similar.

**Figure 7:**
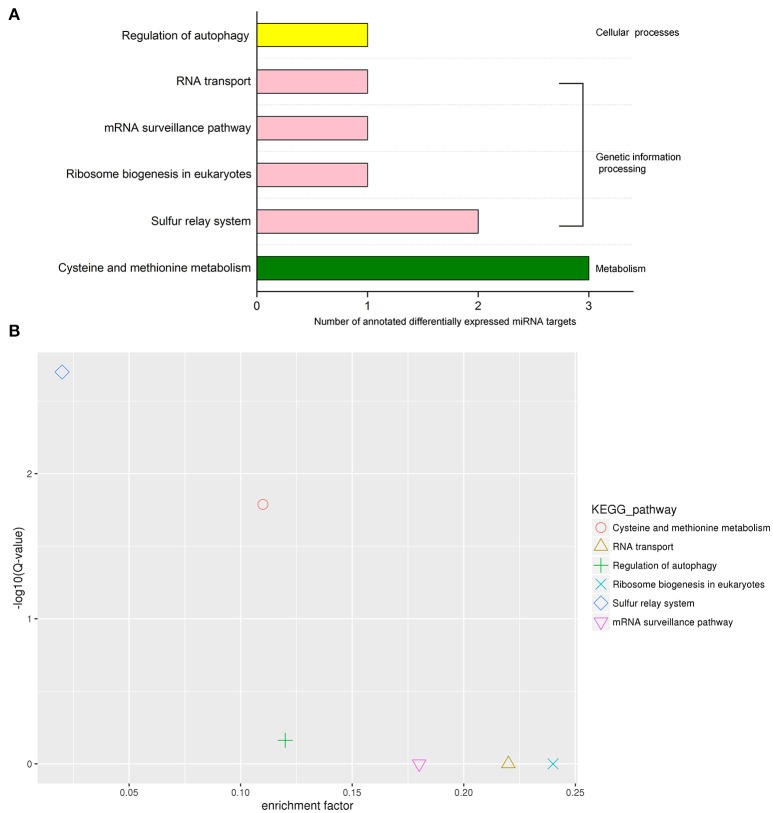
KEGG pathway analysis of differentially expressed miRNA targets. **(A)** KEGG classification of differentially expressed miRNA targets. **(B)** KEGG pathway enrichment scatter diagram of target genes of differentially expressed miRNAs.

Classification of the KEGG pathway and enrichment analysis of the pathway were performed based on KEGG annotation of differentially expressed miRNA targets (Figure 7). The differentially expressed miRNA target genes were annotated into the following six KEGG pathways: cysteine and methionine metabolism, sulfur relay system, RNA transport, ribosome biogenesis in eukaryotes, mRNA surveillance pathway, and autophagy regulation. These six pathways can be classified into three categories, namely, metabolism, genetic information processing, and cellular processes. The pathway with the largest number of annotated target genes was cysteine and methionine metabolism, followed by sulfur relay system (Figure 7A). KEGG pathway enrichment analysis showed that the two pathways with the highest enrichment significance were sulfur relay system and cysteine and methionine metabolism (Figure 7B). We screened four KEGG pathways that may be involved in protein synthesis and found differentially expressed miRNA target gene-related enzymes from the pathway maps: 3-mercaptopyruvate sulfurtransferase (MPST) in cysteine and methionine metabolism, and aminocyclopropane carboxylate oxidase, RNA-binding protein Musashi in mRNA surveillance pathway, and nonsense-mediated mRNA decay protein 3 in ribosome biogenesis in eukaryotes and RNA transport.

### RT-qPCR analysis of miRNA target genes

**Figure 8:**
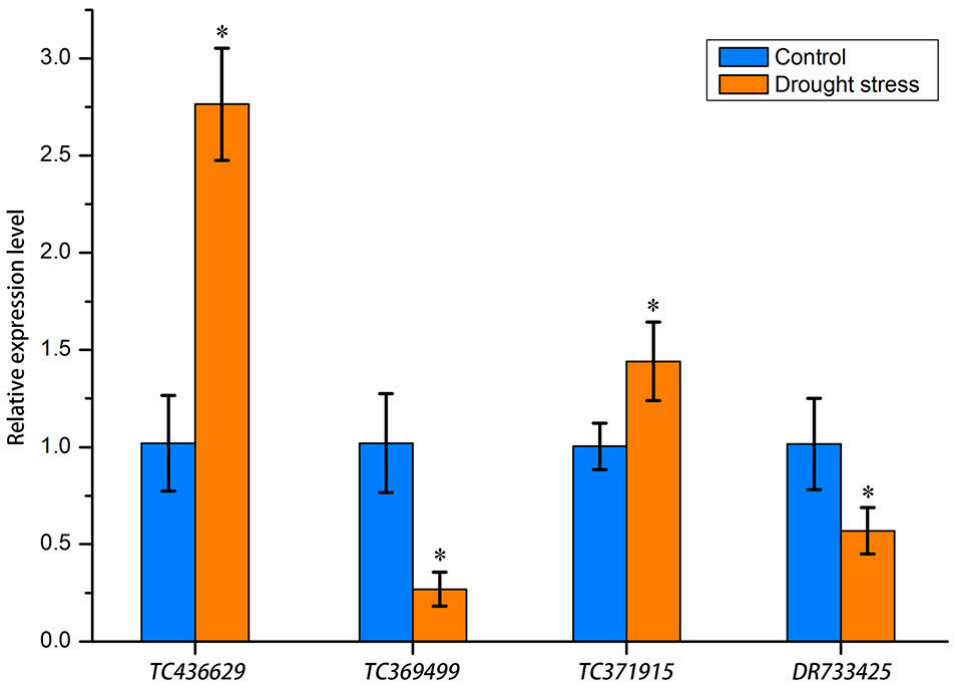
Relative expression levels of four miRNA target genes determined by RT-qPCR. Gene expression levels were normalized against the internal control gene *Ta54825*. Asterisks between the control and drought stress indicate significant difference at *p* < 0.05 as determined by *t*-test. Means were calculated from three replicates.

To further understand the expression patterns of miRNA target genes, we implemented RT-qPCR to determine gene relative expression levels. We selected one corresponding target gene for each differentially expressed miRNA. The selected genes were *TC436629, TC369499, TC371915*, and *DR733425*, which encode proline-rich receptor, 1-aminocyclopropane-1-carboxylate oxidase, MPST and vesicle-associated protein, respectively (bold values in Table 4). The relative expression levels of these genes are shown in Figure 8. Drought stress increased the relative expression levels of *TC436629* and *TC371915* and decreased the relative expression levels of *TC369499* and *DR733425*. Compared with the expression tendency of differentially expressed miRNA in Figure 5, three miRNA-target gene pairs showed opposite trends, defined as negative miRNA regulation. These miRNAs were tae-nsmR10, tae-nsmR5/tae-nsmR6, and tae-miR9654a-3p.

### Accumulation of protein bodies and protein content in wheat endosperm under drought stress

**Figure 9:**
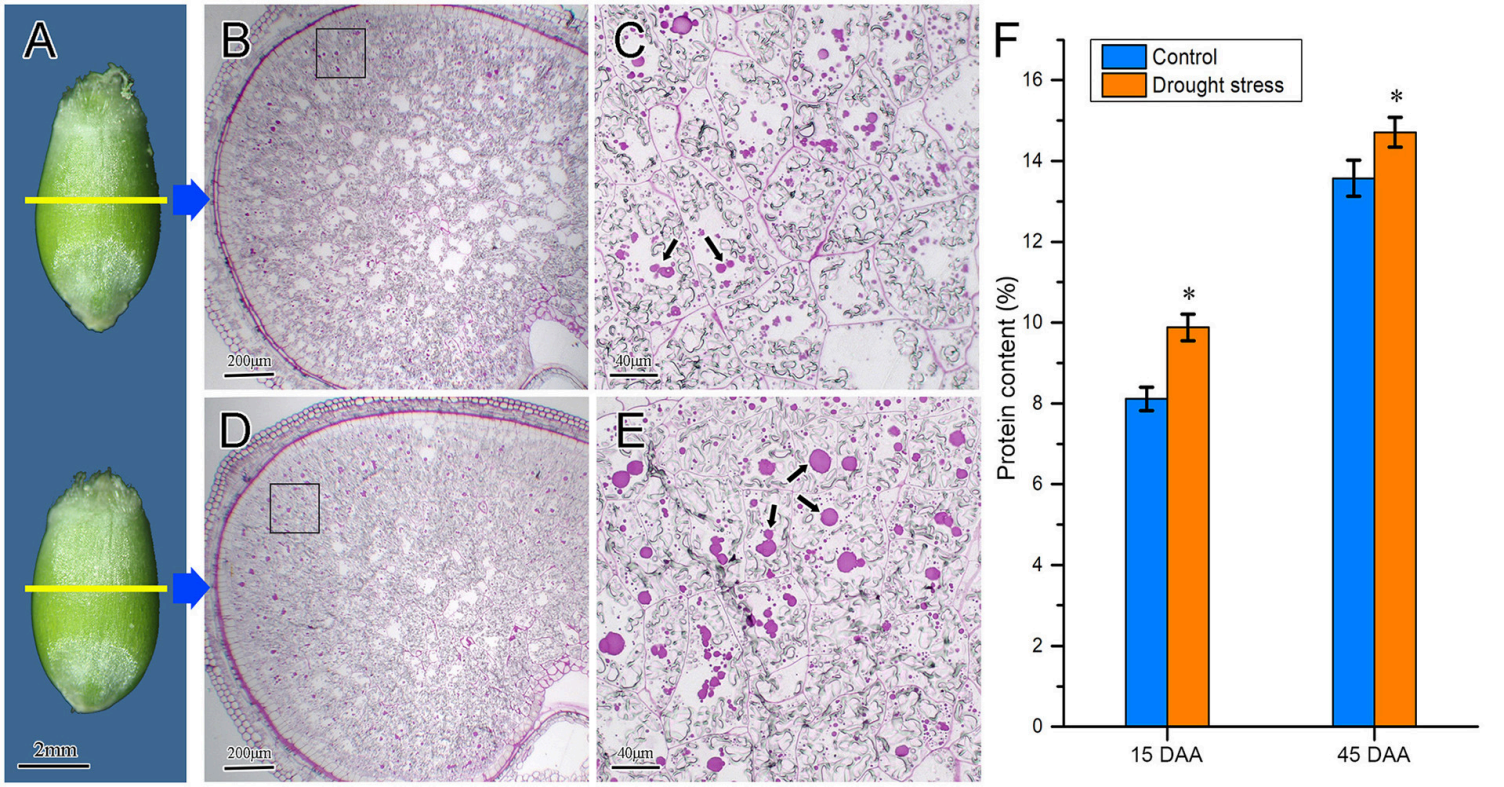
Difference of protein body accumulation in wheat endosperm under control and drought stress conditions. **(A)** Wheat caryopsis profile. The caryopses at the top and the bottom represent control and drought stress conditions, respectively. **(B,C)** Microstructure of wheat endosperm under control condition; **(D,E)** microstructure of wheat endosperm under drought stress. Subpanels **(C,E)** are high-magnification views of the black boxes in subpanels **(B,D)**, respectively. Black arrows in the figures indicate protein bodies. **(F)** Protein contents of wheat caryopsis at 15 and 45 DAA under drought stress. Data are expressed as means from three individual biological replicates. Asterisks above the histogram indicate a significant difference at *p* < 0.05 as determined by *t*-test. Scale bars: **(A)** 2 mm, **(B,D)** 200 μm, and **(C,E)** 40 μm.

The accumulation characteristics of protein bodies in wheat endosperm under drought stress were observed using the semi-thin slice technique. Starch and proteins stored in the amyloid and PB, respectively, are the main reserve substances in wheat endosperm. At 15 DAA, there were no remarkable changes in caryopsis profiles (Figure 9A) and endosperm cells were substantially enriched by starch granules and protein bodies. The protein bodies mainly accumulated in the vicinity of the aleurone layer (Figures 9B,D). Some small protein bodies were observed in endosperm cells under the control condition, whereas large protein aggregations were observed in the endosperm cells of drought-stressed wheat (Figures 9C,E). These findings indicated that drought stress increased the accumulation of protein bodies in the endosperm. Furthermore, we determined the protein content in wheat caryopsis at 15 and 45 DAA (Figure 9F). The results showed that protein contents were significantly enhanced by drought stress at these two stages.

## Discussion

Drought stress is one of the important abiotic stress factors that affects wheat yield and grain quality. To cultivate high-yielding crop varieties with improved stress resistance, the complex molecular mechanisms underlying stress response must be thoroughly understood. Several studies have reported that miRNA plays a significant role in plant response to stress (Lu and Huang, 2008; Eldem et al., 2012). Therefore, identifying and manipulating some abiotic stress-related miRNA regulatory molecules may facilitate improvement in stress resistance and grain yield in cereals (Hikmet et al., 2015), such as wheat (Qu et al., 2015). To date, the methods for studying miRNA are mainly bioinformatics-based prediction and experimentation. Different research methods can be selected for different research purposes.

In recent years, high-throughput sequencing of small RNA has been used to analyze the specific and differential expression of miRNA in plants at different developmental stages, in different tissues, or under different stress treatments. In previous researches, numerous miRNAs were determined in crop species under different stress conditions like barley (Schreiber et al., 2011; Hackenberg et al., 2015) and rice (Yi et al., 2013). Moreover, Ren (2011) identified 21 down-regulated miRNAs and nine up-regulated miRNAs under cold stress in poplar by using the high-throughput sequencing technique. Jian et al. (2010) conducted high-throughput sequencing of five small RNA libraries under drought stress, salt stress, cold stress, and abscisic acid stress, together with a control group. Numerous specifically and differentially expressed miRNAs were found under different stress factors in rice. These studies about miRNA identification based on high-throughput sequencing provide a platform for further analysis of expression profiles of miRNAs and characterization of stress-responsive miRNAs in plants. However, only a few drought responsive miRNAs are known among the miRNAs identified in wheat. Of the 125 miRNAs identified in the present study, only four (tae-nsmR10, tae-csmR5082-1, tae-nsmR5/tae-nsmR6, tae-miR9654a-3p) were found to be differentially expressed under drought stress. Similar results were found in barley which reported that four of 28 miRNAs identified in barley were differentially expressed under dehydration stress conditions (Kantar et al., 2010). The regulation of differentially expressed miRNAs induced by drought may be partly associated with drought-related transcription factors (TFs) such as dehydration-responsive element binding TFs (Morran et al., 2011).

Identifying target genes that are regulated by stress-responsive miRNAs is important for revealing possible roles of miRNA in regulating wheat response to abiotic stress. Based on the high sequence complementarity of mature plant miRNAs and corresponding targets, target genes can be predicted using bioinformatic tools (Ku et al., 2015). Using psRNATarget and BLAST software to predict and annotate putative miRNA targets at the genome level has become one of the most popular approaches (Pandey et al., 2014; Ma et al., 2015). However, the putative target genes need to be further validated by further experiments such as degradome sequencing, which is an effective tool to identify target genes directly cleaved by miRNA on a large scale (German et al., 2008). In the present study, 1,981 target genes were predicted, of which 1,641 target genes were annotated. Quantitative PCR results can reveal the relationship between miRNAs and target genes in stress-responsive expressions. The interaction between miRNA and target genes is complex based on their expression profiles. When a miRNA targets multiple genes, the miRNA only exhibits negative correlation with a certain target gene (Liang et al., 2014). In this study, four differentially expressed miRNAs were identified, and these miRNAs corresponded to 28 target genes. Using RT-qPCR, we analyzed four corresponding target genes of four differentially expressed miRNAs. The results showed that all of them presented different expression profiles. Additionally, three miRNA-target gene pairs (tae-nsmR10–*TC436629*, tae-nsmR5/tae-nsmR6–*TC371915* and tae-miR9654a-3p–*DR733425*) exhibited opposite expression tendencies. This opposite expression tendency has been previously reported (Tang et al., 2014; Wen et al., 2016) and considered as negative regulation of miRNA (Kumar et al., 2015). However, one miRNA-target pair (tae-csmR5082-1–*TC369499*) exhibited similar expression trend, which was inconsistent with the negative regulation theory of miRNA. This may be because the specific miRNA silencing of a certain target gene under drought stress occurs only at a certain development stage or tissue, whereas other target genes remain unaffected.

It is revealed that drought stress can alter expression of many genes and metabolites, including dehydrins, vacuolar acid invertase, glutathione S-transferase (GST), late embryo abundant (LEA), RuBisCo, helicase, proline, and carbohydrates (Nezhadahmadi et al., 2013). As the regulator of gene expression, miRNA is deemed to participate in the regulation of these drought responsive genes. In our study, 28 target genes of four differentially expressed miRNAs were annotated with a wide range of biological functions. Of these, four target genes (*TC390380, CA610228, CA678419, CA646062*) encoded the peroxidase (POD) component. Antioxidant enzymes like POD have the function on elimination of reactive oxygen species and alleviation of drought injury. Wei et al. (2009) reported that down-regulated miR168 and miR528 under drought resulted in up-regulated expression of the target gene mitogen-activated protein kinase (MAPK), which further induced the expression of antioxidant genes and antioxidant enzymes. In addition, the target genes CA663463 and *TC436629* encoded rubisco activase beta and proline-rich receptor, respectively, both of which were related to drought resistance.

In general, miRNA interacts with Argonaute protein to form RNA-induced silencing complexes and specifically bind to the mRNA of target gene by directly cleaving target mRNA or inhibiting protein translation (Carrington and Ambros, 2003), thereby resulting in negative regulation of gene expression at transcriptional and post-transcriptional levels (Guo et al., 2010). The interactions between miRNAs and their target genes through sequence-specific binding provide an accurate regulation pathway for plants to respond to environmental stimuli. Based on KEGG pathway analysis, differentially expressed miRNA targets were found to be enriched in six pathways. Several target genes, which encoded 3-mercaptopyruvate sulfurtransferase (MPST) and aminocyclopropane carboxylate oxidase (ACO), were enriched in cysteine and methionine metabolism pathway, which is an important route for synthesis of sulfur-containing amino acids (Goyer et al., 2007). ACO is known to be involved in ethylene biosynthesis which is related to leaf senescence in drought adaptation (Gómez-Lim et al., 1993). Moreover, nonsense-mediated mRNA decay protein 3 (NMD3) in RNA transport and ribosome biogenesis pathway is also the expression product of differentially expressed miRNA targets, which is key to nuclear export of ribosomal subunits (Chen et al., 2012).

Since the aforementioned pathways were both related to protein biosynthesis, one interpretation of our results is that protein biosynthesis in caryopsis may be regulated by protein synthesis-relevant KEGG pathways. However, the expression level of target genes in these pathways under drought needs further validation. According to gene function analysis, the target of miR9654a-3p is related to vesicle transport of protein in cells. Meanwhile, expression analysis indicated that the up-regulated expression trend of miR9654a-3p was opposite to that of its target gene, which exhibited a down-regulated expression pattern under drought stress. This is predicted to cause decrease in protein output and increase in protein accumulation. These results showed that miR9654a-3p can regulate the expression of coatomer protein-related genes and affect vesicle transport of protein in cells. This may explain previously documented increase in PB accumulation in the endosperm under drought stress. Thus, we concluded that drought stress may affect development of protein bodies in endosperm through the regulation of miRNAs involved in protein synthesis and vesicle transport.

## Conclusion

The present study identified 125 miRNAs in wheat caryopses under control and drought stress conditions, including 110 known miRNAs and 15 putative novel miRNAs. Four differentially expressed miRNAs induced by drought stress may affect the development of protein bodies in caryopsis by regulating the expression levels of target genes involved in protein biosynthesis pathways. These findings offered a putative mechanism underlying the increased accumulation of storage proteins in wheat endosperm under drought stress.

## Author contributions

XC analyzed the data and wrote the paper. YY performed resin slicing and drew the figures. LR accomplished the RNA extraction and RT-qPCR analysis. ZD collected the experimental samples and materials. EZ determined the protein content. XY supervised the experiments. FX designed the experiments and critically revised the paper.

### Conflict of interest statement

The authors declare that the research was conducted in the absence of any commercial or financial relationships that could be construed as a potential conflict of interest.
