# Supplementary material for: Novel Insights into miRNA Regulation of Storage Protein Biosynthesis during Wheat Caryopsis Development under Drought Stress
Source: Front Plant Sci. 2017 Oct 4;8:1707. doi: 10.3389/fpls.2017.01707 (PMC5632728; doi:10.3389/fpls.2017.01707)
Supplement: Supplementary file 2 [file Image1.PDF]

miRBase precursor	: tae-cSMR156-1
Total read count	: 1254
tae-csmR156-1 read count	: 549
tae-miR156 read count	: 549
remaining reads	: 156

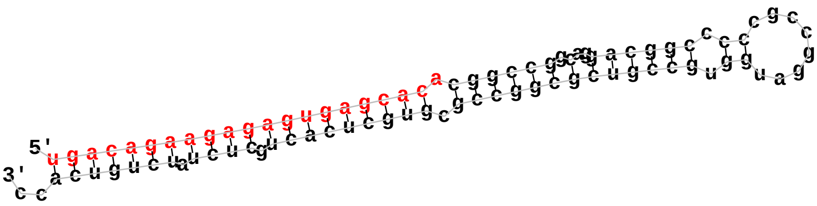

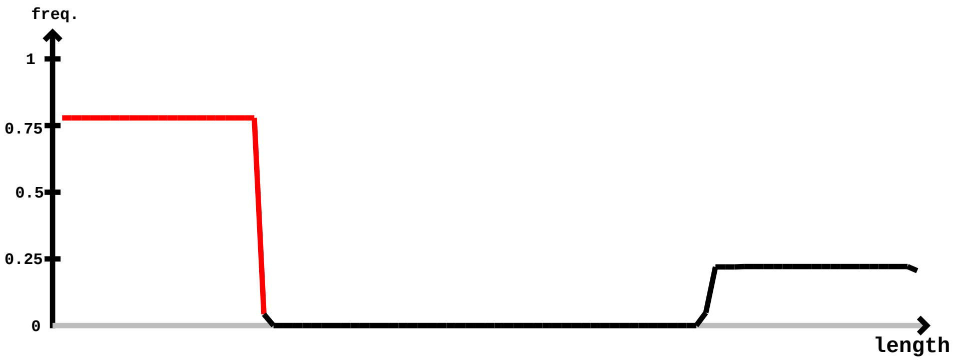


```
     tae-csmR156-1
     tae-miR156
5'-  ugacagaagagagugagcacacggccggcaggacggcccccgccggauggugccgucgcggccgcgugcucacugcucuaucugucacc  -3'   exp
     ((((((((((((((((((.((((((.(..((((((.((........)).)))))))))))))).))))))))).)))).)))))))..   reads    mm    sample
     ugacagaagagagugagcac.....................................................................   211      0     S02
     ugacagaagGgagugagcac.....................................................................   1        1     S02
     ugacagaagagagugGgcac.....................................................................   2        1     S02
     ugacagaagagagugagcaU.....................................................................   2        1     S02
     ugacagaagagagugagcaG.....................................................................   1        1     S02
     ugCcagaagagagugagcac.....................................................................   1        1     S02
     ugacagaGgagagugagcac.....................................................................   1        1     S02
     ugacagCagagagugagcac.....................................................................   1        1     S02
     ugacaUaagagagugagcac.....................................................................   1        1     S02
     ugacagaaUagagugagcac.....................................................................   1        1     S02
     ugacagaUgagagugagcac.....................................................................   1        1     S02
     ugacagaagagagugagcaca....................................................................   11       0     S02
     ugacagaUgagagugagcaca....................................................................   1        1     S02
     ..................................................................ugcucacugcucuaucugucac.   4        0     S02
     ..................................................................ugcucacugcucuaucugAcacc   1        1     S02
     ..................................................................ugcucacugcucuaucugucacc   11       0     S02
     ...................................................................gcucacugcucuaucugucacc   24       0     S02
     ...................................................................Acucacugcucuaucugucacc   1        1     S02
     ...................................................................gcucacugcucuaucugucaGc   36       1     S02

     ugacagaagagagugGgcac.....................................................................   1        1     S01
     ugacagaagagaAugagcac.....................................................................   1        1     S01
     Cgacagaagagagugagcac.....................................................................   2        1     S01
     uAacagaagagagugagcac.....................................................................   1        1     S01
     ugacagaGgagagugagcac.....................................................................   3        1     S01
     ugacagaagagagugagcac.....................................................................   284      0     S01
     ugacagaagagagugagcaU.....................................................................   1        1     S01
     ugacagaagagagCgagcac.....................................................................   1        1     S01
     ugUcagaagagagugagcac.....................................................................   1        1     S01
     ugacaUaagagagugagcac.....................................................................   1        1     S01
     ugacagaagagagugagcacU....................................................................   2        1     S01
     ugacagaagagagugagcGca....................................................................   1        1     S01
     ugacagaagagagugagcaca....................................................................   15       0     S01
     ..................................................................ugcucacugcucCaucugucac.   1        1     S01
```

tae-csmR156-1

tae-miR156

```
ugacagaagagagugagcacacggccggcaggacggcccccgccggauggugccgucgcggccgcgugcucacugcucuaucugucacc

..................................................................ugcucacugcucuaucugucac.    6     0     S01
..................................................................ugcucacugcucuaucugucacc    11    0     S01
...................................................................gcucacugcucuaucugucaGc    24    1     S01
...................................................................gcucacugcucCaucugucacc    1     1     S01
...................................................................gcucacugcucuaucugucacc    33    0     S01
...................................................................gcucacugcucuaucugucacU    2     1     S01
......................................................................cacugcucuaucugucacc    1     0     S01
```

miRBase precursor : tae-cSMR172-1
Total read count : 195
tae-csmR172-1 read count : 93
remaining reads : 102

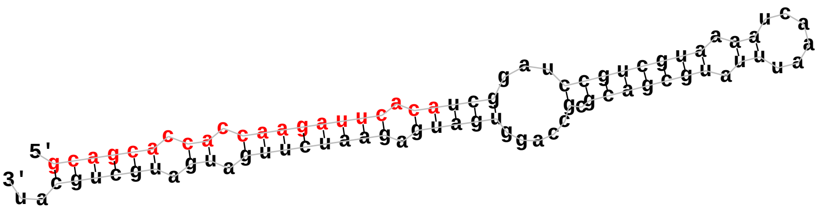

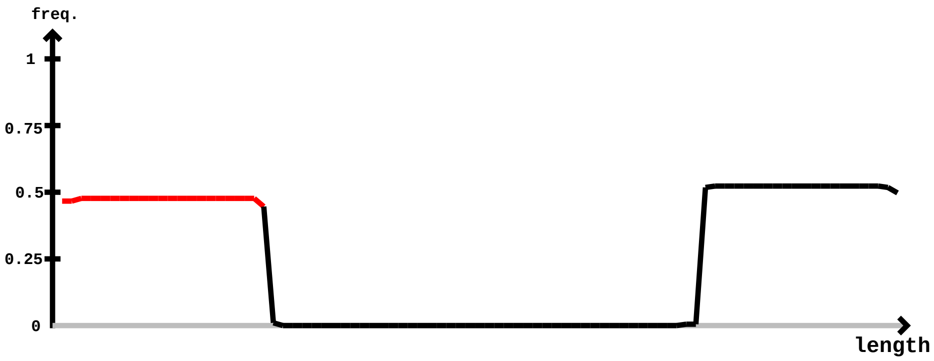

tae-csmR172-1

```
5'- gcagcaccaccaagauucacaucggauccgucguaaaaucaaauuuaugcgacgcgccaggugaugagaaucuugaugaugcugcau  -3'   exp
    ((((((.((.((((((((.(((((...((((((((.((......)).))))))).).....))))).)))))))).)).))))))..   reads   mm   sample
    gcagcaccaccaagauucac...................................................................   1       0    S02
    Acagcaccaccaagauucaca..................................................................   1       1    S02
    gcagcaccaccaagauucaca..................................................................   36      0    S02
    ..................................................................Ggaaucuugaugaugcugca.   1       1    S02
    ..................................................................agaaucuugaugaugcugca.   1       0    S02
    ..................................................................agaaucuugaugaugcugcau   7       0    S02
    ..................................................................agaaucuugaugaugcugcCu   1       1    S02
    ..................................................................Ggaaucuugaugaugcugcau   21      1    S02

    gcagcaccaccaagauucac...................................................................   5       0    S01
    gcagcaccacUaagauucaca..................................................................   1       1    S01
    gcagcaccaccaagauucaca..................................................................   45      0    S01
    gcaAcaccaccaagauucaca..................................................................   1       1    S01
    Acagcaccaccaagauucaca..................................................................   1       1    S01
    .cagcaccaccaagauucacau.................................................................   2       0    S01
    ................................................................ugagaaucuugaugaugcugc..   1       0    S01
    ..................................................................Ggaaucuugaugaugcugca.   2       1    S01
    ..................................................................Ggaaucuugaugaugcugcau   51      1    S01
    ..................................................................agaaucuuAaugaugcugcau   1       1    S01
    ..................................................................agGaucuugaugaugcugcau   1       1    S01
    ..................................................................agaaucuugaugaugcugcau   14      0    S01
    ...................................................................gaaucuugaugaugcugcau   1       0    S01
```

miRBase precursor : tae-cSMR2275-1
Total read count : 304
tae-csmR2275-1 read count : 304
remaining reads : 0

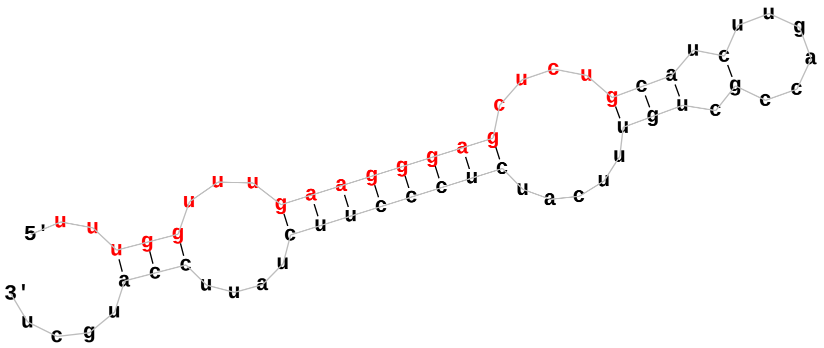

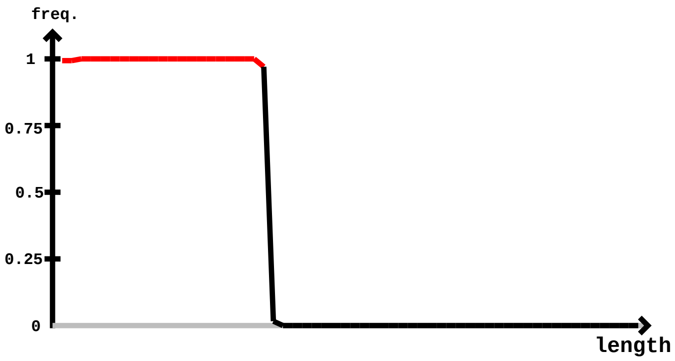

tae-csmR2275-1

```
5'- uuugguuugaagggagcucugcaucuugaccgcuguuucaucucccuucuauuccaugcu   -3'    exp
    ..(((...((((((((....(((.(......).))).....))))))))....)))....     reads      mm         sample
    uuugguuugaagggagcucu........................................     1          0          S02
    Cuugguuugaagggagcucu........................................     2          1          S02
    uuugguuugaagggagcucuA.......................................     1          1          S02
    uuuggGuugaagggagcucug.......................................     37         1          S02
    uuugguuugaagggagcucug.......................................     69         0          S02
    uuugguuugaagUgagcucug.......................................     1          1          S02
    uuuggCuugaagggagcucug.......................................     39         1          S02
    uuugguuugaagggagcucugU......................................     1          1          S02
    uuugguuugaagggagcucugc......................................     1          0          S02

    Cuugguuugaagggagcucu........................................     2          1          S01
    uuuggGuugaagggagcucu........................................     2          1          S01
    uuugguuugaagggagcucu........................................     1          0          S01
    uuugguuugaagggagcucuU.......................................     2          1          S01
    uuugguuugaagggGgcucug.......................................     1          1          S01
    uCugguuugaagggagcucug.......................................     1          1          S01
    uuuggCuugaagggagcucug.......................................     9          1          S01
    uuugguuugaagggagcCcug.......................................     1          1          S01
    uuuggGuugaagggagcucug.......................................     42         1          S01
    uuugguuugaagggagcuAug.......................................     1          1          S01
    uuugguuugaagggagcucug.......................................     85         0          S01
    uuugguuugaagggagcucugU......................................     3          1          S01
    .uuggCuugaagggagcucu........................................     1          1          S01
    .uuggCuugaagggagcucug.......................................     1          1          S01
```

miRBase precursor : tae-cSMR5082-1
Total read count : 110
tae-csmR5082-1 read count : 102
remaining reads : 8

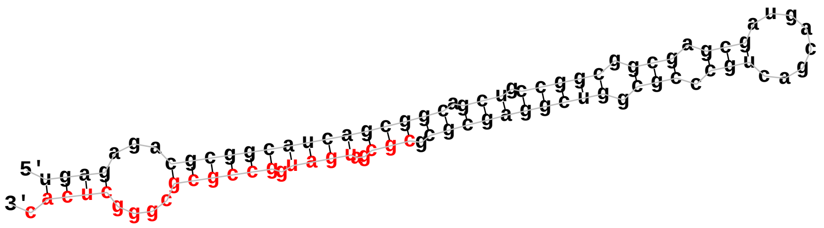

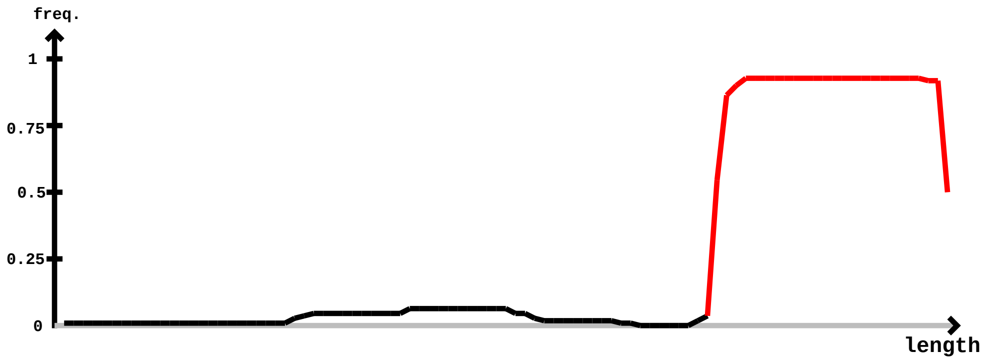


```
                                                                  tae-csmR5082-1
5'- ugagagacgcggcaucagcggcagcugccggcggcgagcgaugacgacugcccgcggucggagcgcgcgcgaugauggccgcgcgggcucac  -3'   exp
    ((((...(((((((((((((((.(((.(((((.(((.(((........))).))).)))))))))).)))..)))).))))))....)))).   reads   mm   sample
    .......................gcugccggcggcgagcgaugacg..............................................   2       0    S01
    .......................gcugccggcggcgagcgaugacgacu...........................................   1       0    S01
    ........................cugccggcggcgagcgaugacgac............................................   1       0    S01
    .........................ugccggcggcgagcgaugacgac............................................   1       0    S01
    .................................................................cgcgcgaugauggccgcgcgggcuca.   1       0    S01
    .................................................................cgcgcgaugauggccgcgcgggcucac   1       0    S01
    ..................................................................gcgcgaugauggccgcgcgggcucac   1       0    S01
    ...................................................................cgcgaugauggccgcgcgggcu...   1       0    S01
    ...................................................................cgcgaugauggccgcgcgggcuca.   17      0    S01
    ...................................................................cgcgaugauggccgcgcgggcucac   29      0    S01
    ....................................................................gcgaugauggccgcgcgggcuca.   10      0    S01
    ....................................................................gcgaugauggccgcgcgggcucac   17      0    S01
    ....................................................................gcgaugauggccgcgcUggcucac   1       1    S01
    .....................................................................cgaugauggccgcgcgggcuca.   4       0    S01
    ......................................................................gaugauggccgcgcgggcuca.   1       0    S01

    ugagagacgcggcaucCgcggca.....................................................................   1       1    S02
    ...................................gagcgaugacgacugcccgcgg...................................   1       0    S02
    ...................................gagcgaugacgacugcccgcgguc.................................   1       0    S02
    ..................................................................gcgcgaugauggccgcgcgggcuca.   1       0    S02
    ...................................................................cgcgaugauggccgcgcgggcuca.   7       0    S02
    ...................................................................cgcgaugauggccgcgcgggcucac   2       0    S02
    ....................................................................gcgaugauggccgcgcgggcuca.   4       0    S02
    ....................................................................gcgaugauggccgcgcgggcucac   3       0    S02
    ......................................................................Uaugauggccgcgcgggcuca.   1       1    S02
    ......................................................................gaugauggccgcgcgggcucac   1       0    S02
```

miRBase precursor : tae-cSMR7743-1
Total read count : 174
tae-csmR7743-1 read count : 160
remaining reads : 14

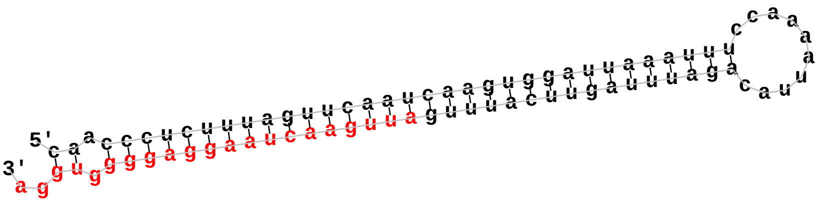

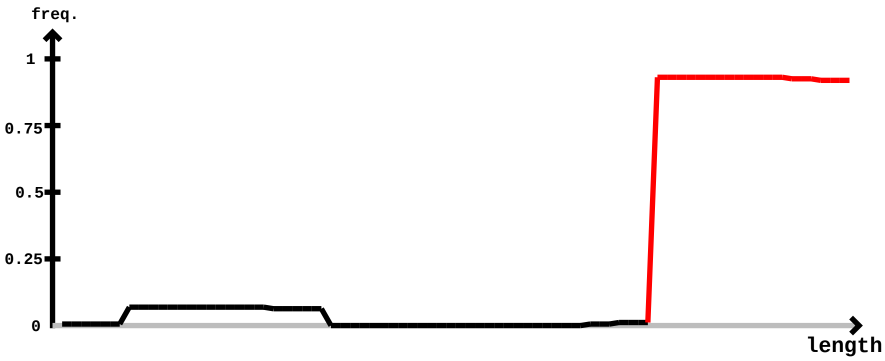


```
                                                                            tae-csmR7743-1
5'- caacccucuuuaguucaaucaaguggauuaaauuuccaaaauuacagauuuaguucauuugauugaacuaaggaggggugga   -3'    exp
    ((.((((((((((((((((((((((((((((((((..........)))))))))))))))))))))))))))))))).))..   reads     mm        sample
    caacccucuuuaguucaauca.............................................................   1         0         S01
    ......ucuuuaguucaaucGagugga.......................................................   1         1         S01
    ......ucuuuaguucaaucaagugga.......................................................   8         0         S01
    ......................................................ucauuugGuugaacuaaggag.......   1         1         S01
    .............................................................auugaacuaaggaggggugg G   1         1         S01
    .............................................................auugaacuaaggGggggugga   1         1         S01
    .............................................................auugaacuaaggaggggugga   85        0         S01
    .............................................................auugaGcuaaggaggggugga   1         1         S01

    ......ucuuuaguucaaucaagugga.......................................................   2         0         S02
    .........................................................uuugauugaacuaaggagggU....   1         1         S02
    .............................................................auugaacuaaggaUgggugga   1         1         S02
    .............................................................auugaacuaaggaggggugga   70        0         S02
    .............................................................auugaacuaGggaggggugga   1         1         S02
```

miRBase precursor : tae-nSMR1
Total read count : 7445
tae-miR9655-3p read count : 1175
tae-nsmR1 read count : 6141
remaining reads : 129

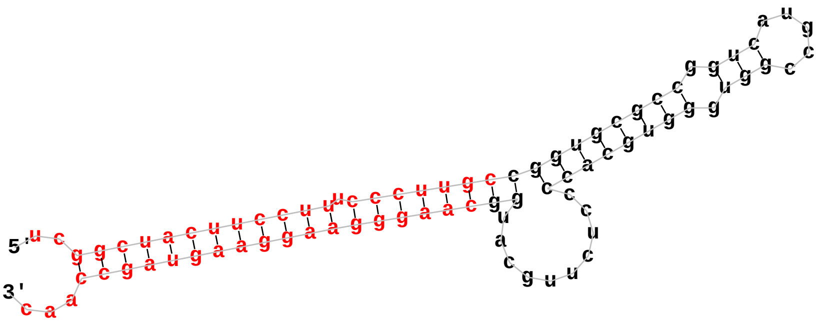

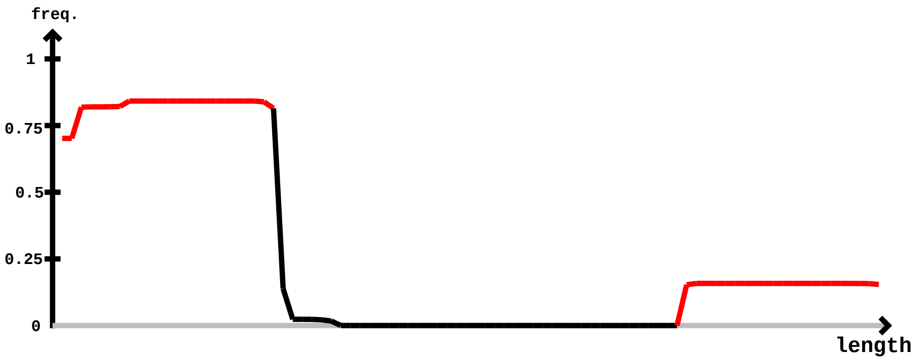

tae-nsmR1

tae-miR9655-3p

```
5'- ucggcuacuuccuuucccuugccggugcgccggucaugccgguggggugcaccccucuugcauggcaagggaaggaaguagccaac  -3'    exp
    ..(((((((((((.((((((((((((((((.(((.....))).))))))))).........)))))))))))))))))))...    reads    mm    sample
    ucggcuacuuccuuuccUuu..................................................................    10       1     S01
    ucggcuacuuccuuucccuC..................................................................    1        1     S01
    ucggcuacuCccuuucccuug.................................................................    1        1     S01
    ucggcuacuucUuuucccuug.................................................................    1        1     S01
    ucggcuacuuccuuucccuug.................................................................    74       0     S01
    ucggcuacuuccuuucccuCg.................................................................    1        1     S01
    ucggcuacuuccuuuUccuug.................................................................    1        1     S01
    ucggcCacuuccuuucccuug.................................................................    1        1     S01
    ucggcuacuuccuCucccuug.................................................................    1        1     S01
    ucggcuacuuccuuucccuuA.................................................................    1        1     S01
    ucgAcuacuuccuuucccuugc................................................................    4        1     S01
    ucggcuacCuccuuucccuugc................................................................    4        1     S01
    ucggcuacuuccuuAcccuugc................................................................    3        1     S01
    ucggcuacuuccuuuUccuugc................................................................    19       1     S01
    Ccggcuacuuccuuucccuugc................................................................    11       1     S01
    ucggcuaAuuccuuucccuugc................................................................    2        1     S01
    ucggcuacuuccuuucccuuCc................................................................    1        1     S01
    ucAgcuacuuccuuucccuugc................................................................    3        1     S01
    ucggcuacuuccuuucccGugc................................................................    2        1     S01
    ucggcuacuuUcuuucccuugc................................................................    2        1     S01
    ucggcuacuuccuuucccuugU................................................................    21       1     S01
    ucggcuacuuccuuuccUuugc................................................................    12       1     S01
    ucCgcuacuuccuuucccuugc................................................................    1        1     S01
    ucggcuacuGccuuucccuugc................................................................    1        1     S01
    ucggcuacuuccuuCccuugc.................................................................    9        1     S01
    ucggcuacuCccuuucccuugc................................................................    5        1     S01
    ucgCcuacuuccuuucccuugc................................................................    1        1     S01
    ucggcuacuuccuuucUcuugc................................................................    3        1     S01
    ucggcuUcuuccuuucccuugc................................................................    3        1     S01
    ucggcuGcuuccuuucccuugc................................................................    1        1     S01
    ucggcuacuuccAuucccuugc................................................................    1        1     S01
    ucggcuacuucUuuucccuugc................................................................    1        1     S01
    Acggcuacuuccuuucccuugc................................................................    2        1     S01
    ucggUuacuuccuuucccuugc................................................................    1        1     S01
```

```
tae-nsmR1
                                                                tae-miR9655-3p
ucggcuacuuccuuucccuugccggugcgccggucaugccggugggugcaccccucuugcauggcaagggaaggaaguagccaac

ucggcuacuuccuuucccCugc...............................................................    6      1    S01
ucggcCacuuccuuucccuugc...............................................................    2      1    S01
ucggcuacuuccuuucccuCgc...............................................................    5      1    S01
uUggcuacuuccuuucccuugc...............................................................    43     1    S01
ucggcuacuuccCuucccuugc...............................................................    6      1    S01
ucggcuacuuccuuucccuugA...............................................................    9      1    S01
ucggcuacuuccuuucccuugc...............................................................    2744   0    S01
ucggcuacAuccuuucccuugc...............................................................    1      1    S01
ucggcuacuuccuuucccAugc...............................................................    2      1    S01
ucggcuacuuccuCucccuugc...............................................................    7      1    S01
ucggcuacuuccuuucccuugcU..............................................................    5      1    S01
ucggcuacuuccuuucccuugcc..............................................................    6      0    S01
ucggcuacuuccuuucccuugcA..............................................................    2      1    S01
ucggcuacuuccuuucccuugccggG...........................................................    1      1    S01
.Uggcuacuuccuuucccuug................................................................    1      1    S01
.Uggcuacuuccuuucccuugc...............................................................    27     1    S01
.cggcuacuuccuuucccuugcA..............................................................    1      1    S01
.Uggcuacuuccuuucccuugcc..............................................................    370    1    S01
.cggcuacuuccuuucccuugcc..............................................................    172    0    S01
.cggcuacuuccuuuccUuugcc..............................................................    1      1    S01
.cggcuacuuccuuAcccuugcc..............................................................    1      1    S01
.cggcuacuuUcuuucccuugcc..............................................................    1      1    S01
.Gggcuacuuccuuucccuugcc..............................................................    1      1    S01
.cggcuacuuccCuucccuugcc..............................................................    1      1    S01
.Uggcuacuuccuuucccuugccggu...........................................................    5      1    S01
.cggcuacuuccuuucccuugccgguA..........................................................    1      1    S01
..Ugcuacuuccuuucccuugcc..............................................................    1      1    S01
..ggcuacuuccuuucccuugcc..............................................................    4      0    S01
...gcuacuuccuuucccuugcUgg............................................................    1      1    S01
....cuacuuccuuucccuugccgguU..........................................................    1      1    S01
.....uacuuccuuucccuugccggu...........................................................    1      0    S01
.....uacuuccuuucccuugccggug..........................................................    2      0    S01
......acuuccuuucccuugccggu...........................................................    1      0    S01
......acuuccuuucccuugccggug..........................................................    11     0    S01
......acuuccuuucccuugccgAug..........................................................    1      1    S01
......acuuccuuucccuugccggugc.........................................................    74     0    S01
......acuuccuuucccuugccggugA.........................................................    1      1    S01
......Ccuuccuuucccuugccggugc.........................................................    1      1    S01
......acuCccuuucccuugccggugc.........................................................    1      1    S01
......acuuccuuucccuugccggugcg........................................................    1      0    S01
............................................................auggcaagggaaggaaguagc....    1      0    S01
................................................................caagggaaggaaguagccaa.    7      0    S01
................................................................cGagggaaggaaguagccaac    1      1    S01
................................................................caagggaaggaaguagcUaac    1      1    S01
................................................................caaggUaaggaaguagccaac    1      1    S01
................................................................caagggaaAgaaguagccaac    1      1    S01
................................................................caagggaaggaaguagccaaA    1      1    S01
................................................................caagggaaggaaguGgccaac    1      1    S01
................................................................caagggaaggaaguagccaGc    3      1    S01
................................................................caagUgaaggaaguagccaac    3      1    S01
................................................................caGggaaggaaguagccaac     2      1    S01
................................................................caagggaaggaaguagccaac    493    0    S01
................................................................caagggaaggaaguagccaaU    3      1    S01
................................................................caagggaaUgaaguagccaac    1      1    S01
................................................................Uaagggaaggaaguagccaac    1      1    S01
................................................................caagggGaggaaguagccaac    1      1    S01
................................................................caaggAaaggaaguagccaac    1      1    S01
................................................................caagggaaggaaguagcAaac    1      1    S01
................................................................caCgggaaggaaguagccaac    1      1    S01
.................................................................aagggaaggaaguagccaa.    4      0    S01
.................................................................aagggaaggaaguagccaac    9      0    S01

ucggcuacuuccuuuccUuu.................................................................    11     1    S02
ucggcuacuuUcuuucccuug................................................................    1      1    S02
uUggcuacuuccuuucccuug................................................................    2      1    S02
ucggcuacuuccuuucccAug................................................................    1      1    S02
Ccggcuacuuccuuucccuug................................................................    1      1    S02
ucggcuacuuccuuucccuug................................................................    93     0    S02
ucggcuacuuccCuucccuugc...............................................................    6      1    S02
```

tae-nsmR1

tae-miR9655-3p

ucggcuacuuccuuucccuugccggugcgccggucaugccggugggugcaccccucuugcauggcaagggaaggaaguagccaac

ucggcuGcuuccuuucccuugc...	1	1	S02
ucggUuacuuccuuucccuugc...	3	1	S02
ucggcuacuuccAuucccuugc...	3	1	S02
ucggcuacuuccuuucccuugc...	1939	0	S02
ucggcuacuuccuuucccuCgc...	1	1	S02
ucggcuacuucAuuucccuugc...	1	1	S02
ucggcuaGuuccuuucccuugc...	1	1	S02
ucggcuUcuuccuuucccuugc...	1	1	S02
ucggcuacuuccuuuccUuugc...	3	1	S02
ucggcuacuuccuuucccuuAc...	1	1	S02
ucggcuacuucUuuucccuugc...	1	1	S02
ucggcuacuuccuuuUccuugc...	10	1	S02
Ccggcuacuuccuuucccuugc...	4	1	S02
ucgAcuacuuccuuucccuugc...	2	1	S02
ucggcuacuuccuuucccuugA...	6	1	S02
ucggcuacuuAcuuucccuugc...	1	1	S02
ucggcuaUuuccuuucccuugc...	2	1	S02
ucAgcuacuuccuuucccuugc...	1	1	S02
ucgUcuacuuccuuucccuugc...	1	1	S02
ucggcuacuuGcuuucccuugc...	1	1	S02
ucggcuacuuUcuuucccuugc...	2	1	S02
uUggcuacuuccuuucccuugc...	19	1	S02
ucggcuacuuccuuucccCugc...	2	1	S02
Acggcuacuuccuuucccuugc...	1	1	S02
ucggcuacCuccuuucccuugc...	7	1	S02
ucggcuacuuccuCucccuugc...	3	1	S02
ucggcuacuuccuuucccuuCc...	2	1	S02
ucggcuacuuccuuucccuugG...	1	1	S02
ucggcuacuuccuuucccuugU...	14	1	S02
ucggcCacuuccuuucccuugc...	2	1	S02
ucggcuacuuccuuCcccuugc...	5	1	S02
ucggcuacuuccuuAcccuugc...	1	1	S02
ucggcuacuCccuuucccuugc...	3	1	S02
ucggcuacuuccuuucUcuugc...	5	1	S02
ucggcuacuuccuuucccuugcc..	8	0	S02
ucggcuacuuccuuucccuugcU..	4	1	S02
ucggcuacuuccuuucccuugcA..	3	1	S02
ucggcuacuuccuuucccuugcG..	1	1	S02
ucggcuacuuccuuucccuugcGgg..	1	1	S02
.Uggcuacuuccuuucccuug..	1	1	S02
.Uggcuacuuccuuucccuugc...	14	1	S02
.cggcuacuuccuuucccuugcc..	95	0	S02
.Uggcuacuuccuuucccuugcc..	170	1	S02
.cggcuacuuccuuucccuugcU..	3	1	S02
.cggcuacAuccuuucccuugcc..	1	1	S02
.cggcuacuuccCuucccuugcc..	1	1	S02
.Uggcuacuuccuuucccuugccggu...	4	1	S02
..ggcuacuuccuuucccuugcc..	3	0	S02
....cuacuuccuuucccuugccggu...	1	0	S02
.....uacuuccuuucccuugccggug..	5	0	S02
.....uacuuccuuucccuugccggugU...	1	1	S02
......acuuccuuuccUuugccggug..	1	1	S02
......acuuccuuucccuCgccggug..	1	1	S02
......acuuccuuucccuugccggug..	10	0	S02
......acuuccuuucccuugccggugc...	44	0	S02
......Gcuuccuuucccuugccggugc...	1	1	S02
......acuuccuuCcccuugccggugc...	1	1	S02
......acuuccCuucccuugccggugc...	1	1	S02
......acuuccuuucccuugccggugcg..	1	0	S02
.............................ccggucaugccggugggugcacc.................................	1	0	S02
....caagggaaggaaguagcca..	3	0	S02
....caagAgaaggaaguagccaa.	1	1	S02
....caagggaaggaaguagccaa.	12	0	S02
....caagggaaggaaguagccaaU	6	1	S02
....caagggaaggaaguagccaac	571	0	S02
....caagggaaggaaguagccaGc	1	1	S02
....caagggaaggaaguagUcaac	1	1	S02
....caagggaaggaaguGgccaac	1	1	S02
....caagggaaggaaguagccaCc	4	1	S02

```
tae-nsmR1
                                                                tae-miR9655-3p
ucggcuacuuccuuucccuugccggugcgccggucaugccggugggugcaccccucuugcauggcaagggaaggaaguagccaac

................................................................caagggaaggaaguagccUac    1    1    S02
................................................................caagggGaggaaguagccaac    1    1    S02
................................................................caagggaaggGaguagccaac    3    1    S02
................................................................caaUggaaggaaguagccaac    1    1    S02
................................................................caagggaaggaCguagccaac    1    1    S02
................................................................Uaagggaaggaaguagccaac    1    1    S02
................................................................caagggaagUaaguagccaac    1    1    S02
................................................................caagggaaggaaguagccGac    2    1    S02
................................................................caagggaUggaaguagccaac    1    1    S02
................................................................caGgggaaggaaguagccaac    1    1    S02
................................................................caagggaaggaagCagccaac    1    1    S02
.................................................................aagggaaggaaguagccaa.    1    0    S02
.................................................................aagggaaggaaguagccaac    23   0    S02
.................................................................aagggaaggaGguagccaac    1    1    S02
```

miRBase precursor : tae-nSMR2
Total read count : 4266
tae-nsmR2 read count : 4265
remaining reads : 1

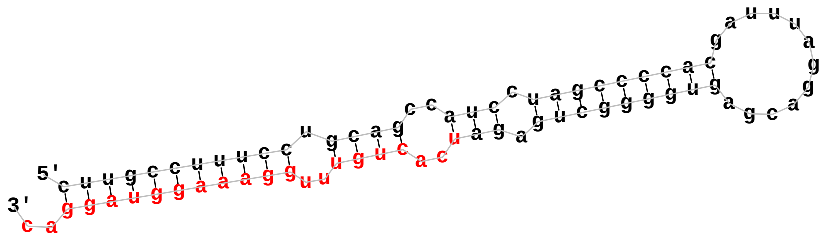

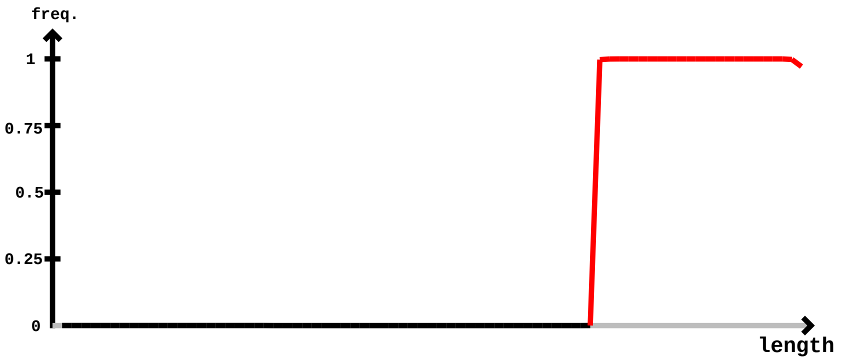


```
                                                          tae-nsmR2
5'- cuugccuuuccugcagccauccuagccccacgauuuaggacgaguggggcugagaucacuguuuggaaagguaggac  -3'   exp
    (((((((((((.((((..(((.(((((((((............))))))))).)))..))))..)))))))))))..    reads    mm     sample
    ......................................................aucacuguuuggaaagguagga.    1        0      S01
    .......................................................ucacuguuuggaaUgguagg..    1        1      S01
    .......................................................ucacuguuuggaaagguagg..    2        0      S01
    .......................................................ucacuguuuggaaUgguagga.    27       1      S01
    .......................................................ucacuguuuggaaagguagga.    36       0      S01
    .......................................................ucacuguuuggaaagguGgga.    1        1      S01
    .......................................................ucacuguuuggaaagguUggac    1        1      S01
    .......................................................ucacuguuuggaaagguaggaU    15       1      S01
    .......................................................ucacuguuuggaaagguaggac    218      0      S01
    .......................................................ucacCguuuggaaagguaggac    2        1      S01
    .......................................................ucacuguuCggaaagguaggac    2        1      S01
    .......................................................ucaUuguuuggaaagguaggac    1        1      S01
    .......................................................ucacuguuuggaaagguaggaA    6        1      S01
    .......................................................ucacuguuuggaaagguaggGc    2        1      S01
    .......................................................uUacuguuuggaaagguaggac    1        1      S01
    .......................................................ucGcuguuuggaaagguaggac    1        1      S01
    .......................................................Ccacuguuuggaaagguaggac    1        1      S01
    .......................................................ucacuguuuggaaCgguaggac    1        1      S01
    .......................................................ucacuguuuggaaagguaggUc    2        1      S01
    .......................................................ucacuguuuggaaUgguaggac    1895     1      S01
    ........................................................cacuguuuggaaUgguaggac    7        1      S01
    ........................................................cacuguuuggaaagguaggac    1        0      S01

    cCugccuuuccugcagccauc........................................................    1        1      S02
    .......................................................ucacuguuuggaaUgguagg..    5        1      S02
    .......................................................ucacuguuuggaaagguGgga.    1        1      S02
    .......................................................ucacuguuuggaaagguagga.    16       0      S02
    .......................................................ucacuguuuggaaUgguagga.    32       1      S02
    .......................................................ucacugCuuggaaagguaggac    1        1      S02
    .......................................................ucacuguuuggaaagguaggaU    4        1      S02
    .......................................................ucacuguuuggGaagguaggac    2        1      S02
    .......................................................ucacuguuuggaGagguaggac    1        1      S02
    .......................................................ucacuguuuggaaCgguaggac    1        1      S02
    .......................................................ucacuguuuggaaagguaUgac    1        1      S02
    .......................................................ucacuUuuuggaaagguaggac    2        1      S02
```

tae-nsmR2

```
cuugccuuuccugcagccauccuagccccacgauuuaggacgaguggggcugagaucacuguuuggaaagguaggac
.......................................................ucacuguuuggaaagguaggaA    5       1       S02
.......................................................ucacuguuuggaaUgguaggac    1802    1       S02
.......................................................ucacuguuuggaaGgguaggac    1       1       S02
.......................................................ucacuguuuggaaagguaggac    163     0       S02
.......................................................ucGcuguuuggaaagguaggac    1       1       S02
........................................................cacuguuuggaaUgguaggac    2       1       S02
.........................................................acuguuuggaaUgguaggac    1       1       S02
```

miRBase precursor : tae-nSMR3
Total read count : 3197
tae-nsmR3 read count : 3092
remaining reads : 105

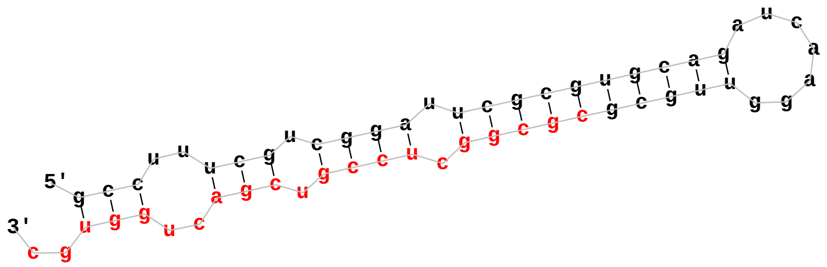

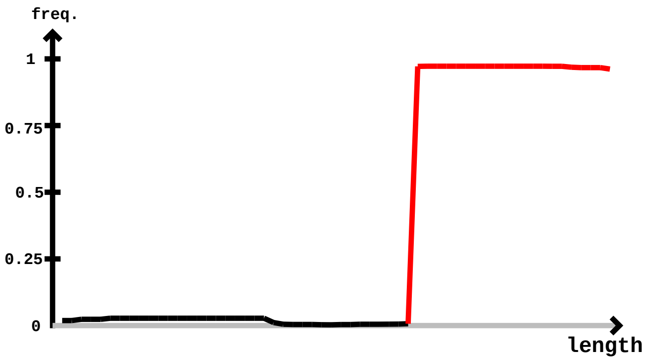


```
                                        tae-nsmR3
5'- gccuuucgucggauucgcgugcagaucaagguugcgcgcggcuccgucgacuggugc  -3'    exp
    (((..(((.((((.((((((((((.......)))))))))).)))).))).)))..   reads      mm         sample
    gccuuucgucggauucgcgug....................................   30         0          S01
    gccuuucgucggauucgcgugA...................................   1          1          S01
    gccuuucgucggauucgcgugc...................................   2          0          S01
    gccuuucgucggauucgcgugca..................................   3          0          S01
    .ccuuucgucggauucgcgugc...................................   7          0          S01
    .ccCuucgucggauucgcgugc...................................   1          1          S01
    ....uucgucggauucgcgugcaAau...............................   3          1          S01
    ....uucgucggauucgcgugcagauc..............................   1          0          S01
    ....uucgucggauucgcgugcaAaucaa............................   2          1          S01
    ....uucgucggauucgcgugcagaucaa............................   2          0          S01
    ............................agguugcgcgcggcuccgucga.......   1          0          S01
    ............................agguugcgcgcggcuccgucgacu.....   1          0          S01
    ............................agguugcgcgcggcuccgucgacug....   2          0          S01
    .............................gguAgcgcgcggcuccgucgacu.....   1          1          S01
    .............................gguugcgcgcggcuccgucgacu.....   4          0          S01
    ..............................guAgcgcgcggcuccgucgacu.....   1          1          S01
    ...................................gcgcggcuccgucgacuggug.   1          0          S01
    ....................................cgcggcuccgucgacuggug.   7          0          S01
    ....................................cgcggcucUgucgacuggugc   2          1          S01
    ....................................cgcggcuccgucgacuggugc   1665       0          S01
    ....................................cgcggcuccgucgacCggugc   3          1          S01
    ....................................cgcgAcuccgucgacuggugc   1          1          S01
    ....................................cgcUgcuccgucgacuggugc   1          1          S01
    ....................................cgcggcuccgucgacugguAc   2          1          S01
    ....................................cgcggcuccgucgacuggCgc   3          1          S01
    ....................................cgUggcuccgucgacuggugc   1          1          S01
    ....................................cgcggcuccgCcgacuggugc   2          1          S01
    ....................................cgcggcuccgucgacuggugU   18         1          S01
    ....................................cgcgUcuccgucgacuggugc   2          1          S01
    ....................................cgcggcuccgucgacuUgugc   1          1          S01
    ....................................cgcggcuccgucgGcuggugc   2          1          S01
    ....................................cgcggcuccgucAacuggugc   2          1          S01
    ....................................cgcggcuUcgucgacuggugc   1          1          S01
    ....................................cAcggcuccgucgacuggugc   3          1          S01
    ....................................cgcAgcuccgucgacuggugc   1          1          S01
```

tae-nsmR3

```
gccuuucgucggauucgcgugcagaucaagguugcgcgcggcuccgucgacuggugc
....................................cUcggcuccgucgacuggugc    3     1     S01
....................................cgcggcuccgGcgacuggugc    2     1     S01
....................................cgcggcCccgucgacuggugc    3     1     S01
....................................cgcggcuccUucgacuggugc    1     1     S01
....................................cgcggcuccgucgacugUugc    1     1     S01
....................................cgcggUuccgucgacuggugc    1     1     S01
....................................Ugcggcuccgucgacuggugc    2     1     S01
....................................cgcggcuccgucUacuggugc    1     1     S01
....................................cgcggcuccgucgacuggugA    7     1     S01
....................................cgcggcuccgucgacugAugc    1     1     S01
....................................cgcggcuccguUgacuggugc    1     1     S01

gccuuucgucggauucgcgug....................................    20    0     S02
gccuuucgucggauuAgcgug....................................    1     1     S02
gccuuucgucggauCcgcgug....................................    1     1     S02
gccuuucgucggauucgcgugc...................................    2     0     S02
.ccuuucgucggauucgcgugc...................................    7     0     S02
....uucgucggauucgcgugcaAauca.............................    3     1     S02
....uucgucggauucgcgugcagaucaa............................    1     0     S02
....uucgucggauucgcgugcaAaucaa............................    1     1     S02
............................agguugcgcgcggcuccgucgacu.....    1     0     S02
............................agguugcgcgcggcuccgucgacug....    1     0     S02
.............................gguugcgcgcggcuccgucgacu.....    1     0     S02
..............................guAgcgcgcggcuccgucgacu.....    1     1     S02
..............................guugcgcgcggcuccgucgacug....    2     0     S02
.................................gcgcgcggcucUgucgacu.....    1     1     S02
..................................cgcgcggcuccgucgacuggugc    1     0     S02
...................................gcgcggcuccgucgacuggug.    3     0     S02
....................................cgcggcuccgucgacuggug.    6     0     S02
....................................cgcggcuccgucgacuggugc    1267  0     S02
....................................cgcggcuccgucgacugCugc    1     1     S02
....................................cgcggcuccAucgacuggugc    1     1     S02
....................................cgcggcuccgucgGcuggugc    3     1     S02
....................................cgcggcucUgucgacuggugc    1     1     S02
....................................cgcggcuccgucgacuggCgc    2     1     S02
....................................cgcggcuccgucgacugAugc    3     1     S02
....................................cgcggcuccUucgacuggugc    1     1     S02
....................................cgcggcuccgucAacuggugc    1     1     S02
....................................cgcggcuccgucgacuggugA    9     1     S02
....................................cgcggcuccgucgacuCgugc    1     1     S02
....................................cgcggcuccgucgaUuggugc    1     1     S02
....................................Ugcggcuccgucgacuggugc    6     1     S02
....................................cgcggcuccgucUacuggugc    1     1     S02
....................................cgcggUuccgucgacuggugc    1     1     S02
....................................cgcggcuccgucgacuggugG    1     1     S02
....................................cgcggcAccgucgacuggugc    1     1     S02
....................................cgcggcuUcgucgacuggugc    3     1     S02
....................................cgcggcuAcgucgacuggugc    1     1     S02
....................................cgcggcuccgucgacGggugc    1     1     S02
....................................cgcggcuccguUgacuggugc    2     1     S02
....................................cgcggcCccgucgacuggugc    4     1     S02
....................................cgcggcuccgucgacCggugc    2     1     S02
....................................cgcggcuccgucgacuggugU    17    1     S02
....................................cgUggcuccgucgacuggugc    1     1     S02
....................................cgcggcuccgucgacugUugc    2     1     S02
....................................cgcggcuccgucgacuAgugc    1     1     S02
....................................cgcgUcuccgucgacuggugc    1     1     S02
....................................cgcggcuccgCcgacuggugc    2     1     S02
....................................cgcAgcuccgucgacuggugc    1     1     S02
.....................................gcggcuccgucgacuggugc    2     0     S02
```

miRBase precursor : tae-nSMR4
Total read count : 898
tae-nsmR4 read count : 534
remaining reads : 364

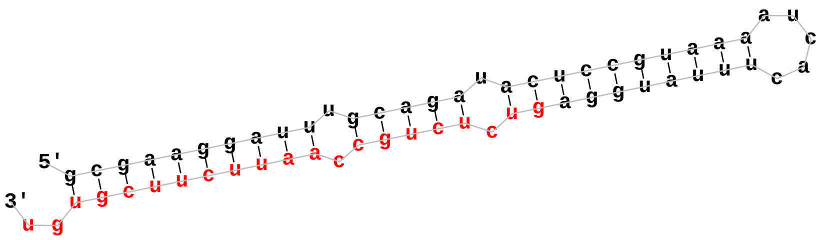

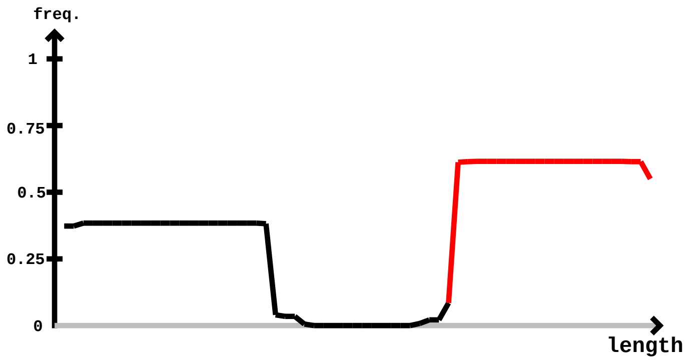


```
                                                         tae-nsmR4
5'- gcgaaggauuugcagauacuccguaaaaucacuuuauggagucucugccaauucuucgugu  -3'   exp
    ((((((((((.(((((.((((((((((.....)))))))))).))))).))))))))))..   reads      mm         sample
    gcgaCggauuugcagauacuc........................................   1          1          S01
    gcgGaggauuugcagauacuc........................................   1          1          S01
    gcgaaggauuugcagauacCc........................................   2          1          S01
    gcgaaggauuugcagauacuA........................................   1          1          S01
    gcgaaggauuugcagauacuc........................................   185        0          S01
    gcgaaggauuugcagauacuUcgu.....................................   1          1          S01
    gcgaaggauuugcagauacuccgu.....................................   13         0          S01
    .cgaaggauuugcagauacucc.......................................   2          0          S01
    .cgaaggauuugcagauacuccgua....................................   3          0          S01
    ....................................uggagucucugccaauucuucg...   1          0          S01
    ....................................uggagucucugccaauucuucguU.   2          1          S01
    ....................................uggagucucugccaauucuucgug.   1          0          S01
    .....................................ggagucucugccaauucuucgugu   8          0          S01
    .....................................ggCgucucugccaauucuucgugu   1          1          S01
    .......................................agucucugccaauucuucgug.   25         0          S01
    .......................................agCcucugccaauucuucgug.   1          1          S01
    .......................................agucucugccaauucuucAug.   1          1          S01
    .......................................agucuUugccaauucuucgug.   1          1          S01
    .......................................agucucugccaauucuucguA.   1          1          S01
    .......................................agucucugccaauucuucgugA   1          1          S01
    .......................................agucucugccaauucuucgugu   3          0          S01
    ........................................gucucugccaauucuCcgugu   1          1          S01
    ........................................Aucucugccaauucuucgugu   2          1          S01
    ........................................gucucugccaauucuucAugu   1          1          S01
    ........................................gucucugccaauCcuucgugu   1          1          S01
    ........................................gucucugccaauucuucgugu   264        0          S01
    ........................................gucucugccaauucCucgugu   2          1          S01
    ........................................gCcucugccaauucuucgugu   1          1          S01
    ........................................gucGcugccaauucuucgugu   1          1          S01
    ........................................gucucugccaaCucuucgugu   2          1          S01
    ........................................gucucCgccaauucuucgugu   1          1          S01
    ........................................gucuUugccaauucuucgugu   1          1          S01
    ........................................gucucugccaauucuucgugA   2          1          S01
    .........................................ucucugccaauucuucgugu   1          0          S01
    ..........................................cucugccaauucuucgugu   1          0          S01
```

tae-nsmR4

```
gcgaaggauuugcagauacuccguaaaaucacuuuauggagucucugccaauucuucgugu

gcgaaggauuugcagauacu.........................................   2     0     S02
gcgaaggauuugcagauGcuc........................................   1     1     S02
gcgaaggauuugcagauUcuc........................................   1     1     S02
gcgaaggauuugcagauacuA........................................   1     1     S02
gcgaaggauuugcagauacuc........................................   112   0     S02
gcgaaggauuugcagauacuU........................................   1     1     S02
gcgaaggauuugUagauacuc........................................   1     1     S02
gcgaaggauuugcagauacuccgu.....................................   12    0     S02
.cgaaggauuugcagauacucc.......................................   3     0     S02
.cgaaggauuugcagauacuccgua....................................   2     0     S02
....................................uggagucucugccaauucuucgug.   3     0     S02
.....................................ggagucucugccaauucuucgugu   3     0     S02
......................................agucucugccaauucuucgug.   23    0     S02
......................................agucucugccaauucuucgugu   1     0     S02
........................................gucCcugccaauucuucgugu   1     1     S02
........................................Aucucugccaauucuucgugu   1     1     S02
........................................Uucucugccaauucuucgugu   1     1     S02
........................................gucucugccaauucuCcgugu   2     1     S02
........................................gucucCgccaauucuucgugu   1     1     S02
........................................gucucugcUaauucuucgugu   1     1     S02
........................................gucucugccaauucuucgugA   1     1     S02
........................................gucucugccaauucuucgCgu   1     1     S02
........................................gucucugccaauucuucgugu   182   0     S02
........................................gucucugccaauucuucgugG   1     1     S02
........................................gucucugccaauucuucguCu   1     1     S02
........................................gucucugccaauucuucgugC   1     1     S02
........................................gucucuUccaauucuucgugu   1     1     S02
.........................................ucucugccaauucuucgugu   1     0     S02
```

miRBase precursor : tae-nSMR5
Total read count : 190
tae-nsmR5 read count : 94
tae-nsmR6 read count : 94
remaining reads : 2

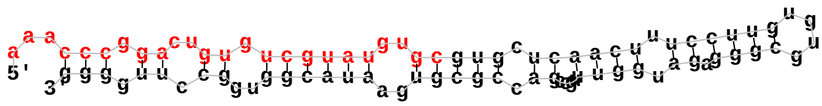

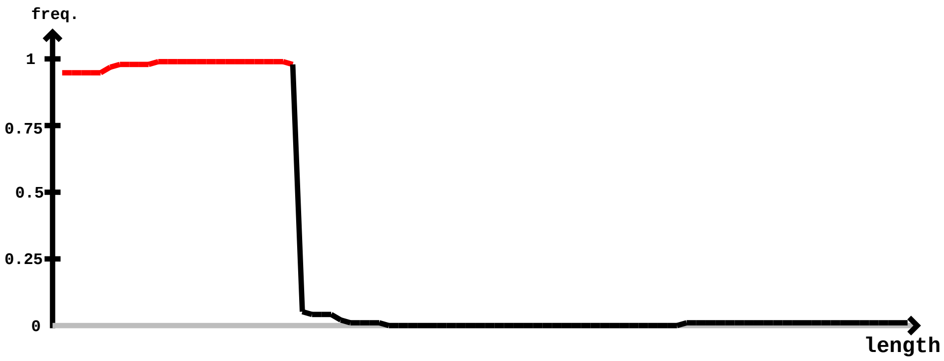


```
     tae-nsmR5
     tae-nsmR6
5'- aaacccggacugugucguaugugcgugcucaacuuuccuugugugcgggagaugguuugccgaccgcgugaauacgguggccuugggg  -3'   exp
    ...(((((((((((..((((((((.((..((((.((((((....)))).)).)))).)))).))))))..)))))).)))))))....))))).   reads   mm   sample
    aaacccggacugugucguaugug.................................................................   1   0   S02
    aaacccggacugugucguaugugc................................................................   19   0   S02
    aaacccggacugugucUuaugugc................................................................   1   1   S02

    Gaacccggacugugucguaugugc................................................................   1   1   S01
    aaaccUggacugugucguaugugc................................................................   1   1   S01
    aaacccggacugugucguaugugc................................................................   65   0   S01
    aaacccggacugugCcguaugugc................................................................   1   1   S01
    aaacccggacugugucguaugugU................................................................   1   1   S01
    aaacccggacugugucguaugugcg...............................................................   1   0   S01
    ....ccggacugugucguaugugcgugc............................................................   2   0   S01
    .....cggacugugucguaugugcgugcu...........................................................   1   0   S01
    .........cugugucguaugugcgugcucaac.......................................................   1   0   S01
    ................................................................cgcgugaauacgguggccuugggg   1   0   S01
```

miRBase precursor	: tae-nSMR6
Total read count	: 204
tae-nsmR5 read count	: 97
tae-nsmR6 read count	: 97
remaining reads	: 10

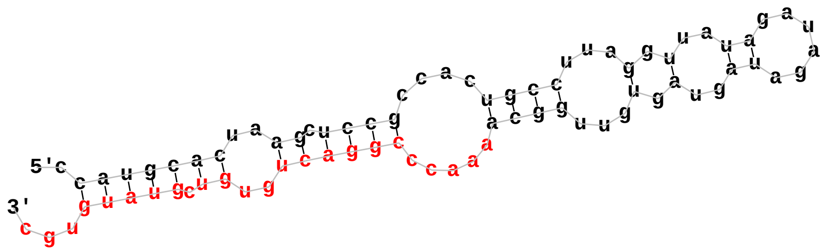

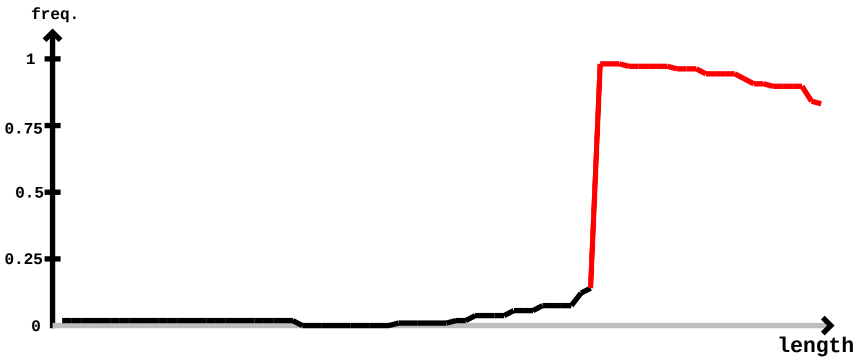


```
                                                                    tae-nsmR5
                                                                    tae-nsmR6
5'- ccaugcacuaagcuccgccacugccuuagguuauagauagauaguaguguuggcaaaacccggacugugucguaugugc  -3'   exp
    .(((((((..((.((((....((((...(.(..((......))..).)...)))).....))))))..)).)))))...   reads     mm        sample
    ccaugcacuaagcucUgccacugc.......................................................   1         1         S02
    ..........................................aguaguguuggcaaaacccggacu.............   2         0         S02
    ..............................................guguuggcaaaacccggacugugu.........   1         0         S02
    .................................................uuggcaaaacccggacuguguc........   2         0         S02
    .....................................................caaaacccggacugugucguaugu..   1         0         S02
    .......................................................aaacccggacugugucguaugug.   1         0         S02
    .......................................................aaacccggacugugucguaugugc   19        0         S02
    .......................................................aaacccggacugugucUuaugugc   1         1         S02

    ccaugcacuaagcucUgccacugc.......................................................   1         1         S01
    ..................................agauagauaguaguguuggcaaaa.....................   1         0         S01
    ........................................auaguaguguuggcaaaacccgg................   1         0         S01
    ..............................................guguuggcaaaacccggacugugu.........   1         0         S01
    .....................................................caaaacccggacugugucguaugu..   4         0         S01
    ......................................................aaaacccggacugugucgu......   1         0         S01
    ......................................................aaaacccggacugugucguaugu..   1         0         S01
    .......................................................aaaccUggacugugucguaugugc   1         1         S01
    .......................................................Gaacccggacugugucguaugugc   1         1         S01
    .......................................................aaacccggacugugCcguaugugc   1         1         S01
    .......................................................aaacccggacugugucguaugugU   1         1         S01
    .......................................................aaacccggacugugucguaugugc   65        0         S01
```

miRBase precursor : tae-nSMR7
Total read count : 428
tae-nsmR7 read count : 427
remaining reads : 1

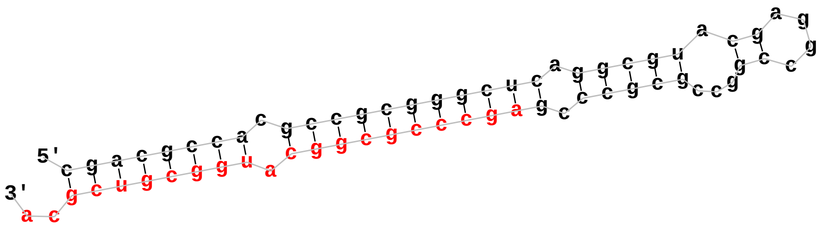

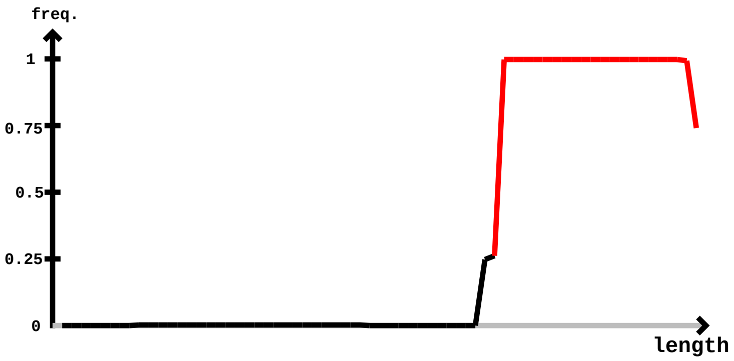


```
                                              tae-nsmR7
5'- cgacgccacgccgcgggcucaggcguacgaggccggccgcgcccgagcccgcggcauggcgucgca   -3'    exp
    ((((((((.(((((((((((.(((((.((....))...))))).))))))))))).))))))))..    reads      mm        sample
    ...........................................cgagcccgcggcauggcgucg..    1          0         S01
    ...........................................cUagcccgcggcauggcgucgc.    1          1         S01
    ...........................................cgagcccgcggcauggcgucgU.    4          1         S01
    ...........................................cgagcccgcggcauggcguUgc.    1          1         S01
    ...........................................cgagcccgcggcauggcgucgc.    54         0         S01
    ...........................................cgagcccgUggcauggcgucgc.    2          1         S01
    ...........................................cgagcccgcggcauggcgucgca    2          0         S01
    ............................................gagcccgcggcauggcgucg..    1          0         S01
    ............................................gagcccgcggcauggcgucgc.    2          0         S01
    .............................................aCcccgcggcauggcgucgca    2          1         S01
    .............................................agcccgcggcauggcgucgca    196        0         S01
    .............................................agcccgcggcaugAcgucgca    1          1         S01
    .............................................agcccgcggcauggcgucgcG    1          1         S01
    .............................................agcccgcgCcauggcgucgca    1          1         S01
    .............................................agcccgcggcauggcgCcgca    1          1         S01

    .......acgccgcgggcucaggcguacgag...................................    1          0         S02
    ...........................................cgagcccUcggcauggcgucgc.    1          1         S02
    ...........................................cgagcccgcggcauggcgucgU.    3          1         S02
    ...........................................Ugagcccgcggcauggcgucgc.    1          1         S02
    ...........................................cgagcccgcggcGuggcgucgc.    1          1         S02
    ...........................................cgagGccgcggcauggcgucgc.    1          1         S02
    ...........................................cgagcccgcggcauggcgucgc.    34         0         S02
    ............................................gagcccgcggcauggcgucgc.    2          0         S02
    ............................................Cagcccgcggcauggcgucgc.    1          1         S02
    .............................................agcccgcggcauggcgucgcG    1          1         S02
    .............................................agcccUcggcauggcgucgca    1          1         S02
    .............................................agcccgcggcauggUgucgca    1          1         S02
    .............................................agcccgcggcauggcgucgca    108        0         S02
    .............................................agcccgcggcauggcgCcgca    2          1         S02
```

miRBase precursor	: tae-nSMR8
Total read count	: 184
tae-nsmR8 read count	: 183
remaining reads	: 1

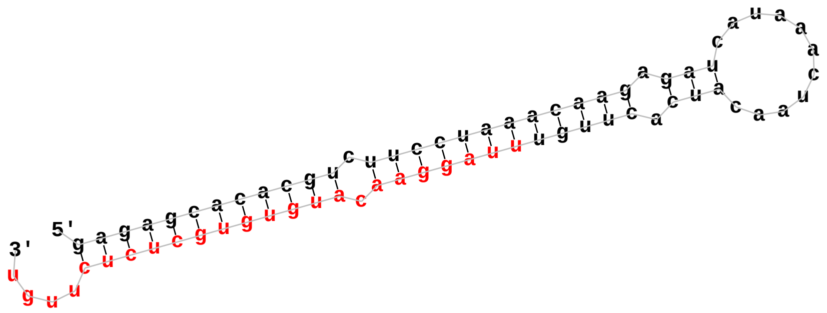

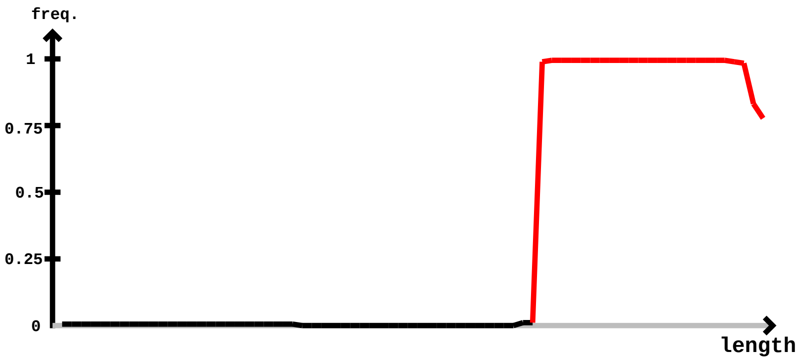


```
                                                   tae-nsmR8
5'- gagagcacacgucuuccuaaacaagagaucauaaacuaacaucacuuguuuaggaacaugugugcucucuugu   -3'    exp
    ((((((((((((.((((((((((((.(((...........))).)))))))))))).))))))))))))....   reads     mm        sample
    ...............................................guuuaggaacaugugugcucucuu..   2         0         S01
    .................................................uuaggaacaugugugcucuc....   1         0         S01
    .................................................uuaggaacaugugugcucucuu..   13        0         S01
    .................................................uuaggaacaugugugcucucCu..   1         1         S01
    .................................................uuaggaacaugugugcucuUuu..   1         1         S01
    .................................................uuaggaacaugugugcCcucuu..   1         1         S01
    .................................................uuaggaacaugugugcucucuug.   4         0         S01
    .................................................uuaggaacaugugugcucucuuU.   4         1         S01
    .................................................uuaggaacaugugugUucucuugu   1         1         S01
    .................................................uuGggaacaugugugcucucuugu   1         1         S01
    .................................................uuaggaacaugugAgcucucuugu   2         1         S01
    .................................................uuaggaacaugugugcucucuugu   80        0         S01
    ..................................................uaggaacaugugugcucucuugu   1         0         S01

    gagagcacacgucuuccCaaacaa.................................................   1         1         S02
    .................................................uuaggaacaugugugcuUucu...   1         1         S02
    .................................................uuaggaacaugugugcucucuu..   9         0         S02
    .................................................uuaggaacaugugugcucucuC..   1         1         S02
    .................................................uuaggaacaugugugcucucuug.   1         0         S02
    .................................................uuaggaacaugugugcucucuuU.   1         1         S02
    .................................................uuaggaacauguguAcucucuugu   2         1         S02
    .................................................uuaggaacaugugugcucucuugu   53        0         S02
    .................................................uuaggaacaugugugcucCcuugu   1         1         S02
    .................................................uuaggaacaugugugcucucuugC   2         1         S02
```

miRBase precursor	: tae-nSMR9
Total read count	: 13615
tae-nsmR9 read count	: 12665
remaining reads	: 950

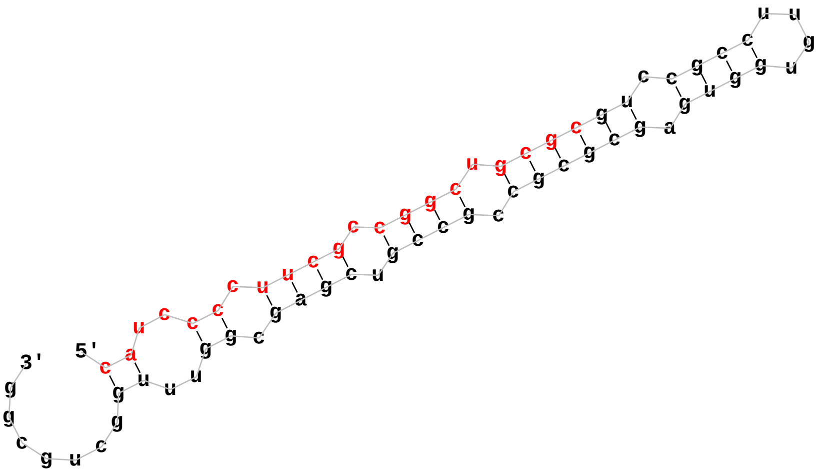

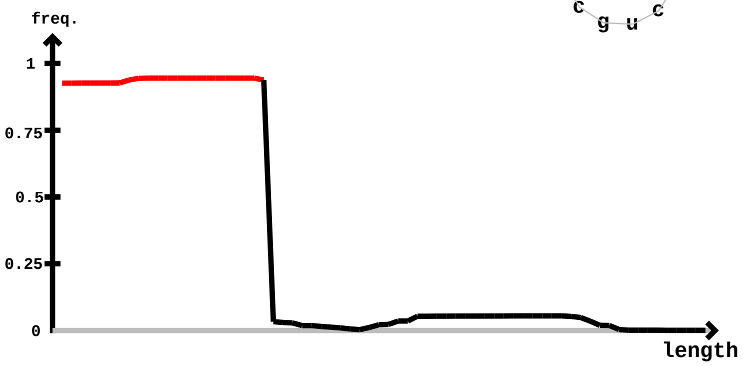


```
     tae-nsmR9
5'-  cauccccuucgccggcugcgcguccgccuuguggugagcgcgccgccgucgagcgguuuggcugcgg   -3'    exp
     ((..((.((((.((((.((((((.((((....)))).)))))).)))).)))).))..)).......    reads       mm          sample
     cauccccuucgccggcugU................................................    1           1           S01
     cauccccuucgccggcugcg...............................................    52          0           S01
     caucccUuucgccggcugcg...............................................    1           1           S01
     cauUcccuucgccggcugcg...............................................    3           1           S01
     cauccAcuucgccggcugcgc..............................................    3           1           S01
     cauccccuCcgccggcugcgc..............................................    16          1           S01
     cauccccuucgccggcugcgG.............................................    1           1           S01
     Uauccccuucgccggcugcgc..............................................    17          1           S01
     cauccccuucgccggcugUgc..............................................    6           1           S01
     cauccccuAcgccggcugcgc..............................................    2           1           S01
     cauccccuucgccggcuCcgc..............................................    1           1           S01
     cauccccuucgccgUcugcgc..............................................    3           1           S01
     cauccccuucgccggcugcgU.............................................    36          1           S01
     cauccccuucAccggcugcgc..............................................    12          1           S01
     cauAcccuucgccggcugcgc..............................................    3           1           S01
     cauccccuucgAcggcugcgc..............................................    2           1           S01
     cauccccuucgccggcugcAc..............................................    7           1           S01
     cauccccuucgccAgcugcgc..............................................    4           1           S01
     cauccccuucgccggcGgcgc..............................................    2           1           S01
     cauccccuuUgccggcugcgc..............................................    4           1           S01
     caCccccuucgccggcugcgc..............................................    12          1           S01
     cauccccuucgccgAcugcgc..............................................    12          1           S01
     caucccGuucgccggcugcgc..............................................    20          1           S01
     cauUcccuucgccggcugcgc..............................................    2           1           S01
     cauccccuucgcAggcugcgc..............................................    3           1           S01
     cauccccuuAgccggcugcgc..............................................    3           1           S01
     cauccccuucgccggcugcgc..............................................    6784        0           S01
     caAccccuucgccggcugcgc..............................................    5           1           S01
     cauccccuucgcGggcugcgc..............................................    1           1           S01
     cauccccuucgccggcAgcgc..............................................    2           1           S01
     cauccccuucgUcggcugcgc..............................................    12          1           S01
     Gauccccuucgccggcugcgc..............................................    4           1           S01
     caucccGucgccggcugcgc...............................................    2           1           S01
     cauccccuucgcUggcugcgc..............................................    9           1           S01
     cUuccccuucgccggcugcgc..............................................    2           1           S01
```

tae-nsmR9

```
cauccccuucgccggcugcgcguccgccuuguggugagcgcgccgccgucgagcgguuuggcugcgg
cauccUcuucgccggcugcgc..............................................    7     1     S01
caucAccuucgccggcugcgc..............................................    1     1     S01
cauccccAucgccggcugcgc..............................................    1     1     S01
cauccccuucgccggcugcgA..............................................    7     1     S01
cauccccuucgccggcugGgc..............................................    3     1     S01
cauccccuucgccCgcugcgc..............................................    1     1     S01
cauccccuucgccggcuUcgc..............................................    5     1     S01
caucUccuucgccggcugcgc..............................................    12    1     S01
cauccccuucgGcggcugcgc..............................................    3     1     S01
cauccccuucgccggAugcgc..............................................    2     1     S01
cauccccuucCccggcugcgc..............................................    1     1     S01
cauccccuucUccggcugcgc..............................................    3     1     S01
caucccUuucgccggcugcgc..............................................    11    1     S01
cauccccuucgccggcugcgcg.............................................    1     0     S01
cauccccuucgccggcugcgcA.............................................    17    1     S01
cauccccuucgccggcugcgcU.............................................    1     1     S01
cauccccuucgccggcugcgcgu............................................    2     0     S01
cauccccuucgccggcugcgcAu............................................    12    1     S01
cauccccuucgcUggcugcgcgu............................................    1     1     S01
caucccGuucgccggcugcgcguc...........................................    1     1     S01
cauccccuucgccggcugcgcguU...........................................    10    1     S01
cauccccuucgccggcugcgcAuc...........................................    57    1     S01
.auUcccuucgccggcugcgcg.............................................    4     1     S01
.....cGuucgccggcugcgcguccg.........................................    2     1     S01
......cuucgccggcugcgcguccA.........................................    1     1     S01
......Guucgccggcugcgcguccg.........................................    15    1     S01
......cuucgccggcugcgcguUcg.........................................    8     1     S01
......cuucgccggcugcgcguUcgc........................................    9     1     S01
......cuucgccggcugcgcguUcgcc.......................................    9     1     S01
......cuucgccggcugcgcguUcgccu......................................    22    1     S01
......Guucgccggcugcgcguccgccu......................................    1     1     S01
......cuucgccggcugcgcguUcgccuu.....................................    10    1     S01
......Guucgccggcugcgcguccgccuu.....................................    3     1     S01
......Guucgccggcugcgcguccgccuug....................................    2     1     S01
......cuucgccggcugcgcguUcgccuug....................................    11    1     S01
.......uucgccggcugcgcguccgc........................................    6     0     S01
.......uucgccggcugcgcguccgU........................................    1     1     S01
.......uucgccggcugcgcguccgcc.......................................    2     0     S01
.......uucgccggcugcgcguUcgcc.......................................    4     1     S01
.......uucgccggcugcgcguccgcU.......................................    3     1     S01
.......uucgccggcugcgcguccgccu......................................    10    0     S01
.......uucgccggcugcgcguccgcUuu.....................................    2     1     S01
.......uucgccggcugcgcguUcgccuu.....................................    3     1     S01
.......uucgccggcugcgcguccgccuu.....................................    13    0     S01
.......uucgccggcugcgcguccgccuug....................................    8     0     S01
.......uucgccggcugcgcguccgccuuU....................................    1     1     S01
........ucgccggcugcgcguccAcc.......................................    4     1     S01
........ucgccggcugcgcguccgccu......................................    1     0     S01
........ucgccggcugcgcguccgccuu.....................................    1     0     S01
...........................cuugCggugagcgcgccgccgucgagc.............    1     1     S01
..............................guggugagcgcgccgccgucgagG.............    1     1     S01
..............................guggugagcgcgccgccgucgagGg............    6     1     S01
...............................uggugagcgcgccgccgucga...............    6     0     S01
...............................Cggugagcgcgccgccgucgag..............    15    1     S01
...............................uggugGgcgcgccgccgucgagc.............    1     1     S01
...............................uggugagcgcgccgccgucgagc.............    1     0     S01
...............................uggugagcgcgccgccgucgagG.............    1     1     S01
...............................Cggugagcgcgccgccgucgagc.............    79    1     S01
...............................uggugagcgcgccgccgucgagGg............    2     1     S01
...............................Cggugagcgcgccgccgucgagcg............    13    1     S01
...............................uggugagcgcgccgccgucgaAcg............    1     1     S01
................................ggugagcgcgccgccgucga...............    15    0     S01
................................ggugagcgcgccgcUgucgag..............    2     1     S01
................................ggugUgcgcgccgccgucgag..............    1     1     S01
................................ggugagcgcgccgccgucgag..............    15    0     S01
................................ggugagcgcgccgccgucgagU.............    4     1     S01
................................ggugagcgcgccgccgucgaAc.............    6     1     S01
................................ggugagcgcgccgccgucgagc.............    17    0     S01
................................ggugGgcgcgccgccgucgagc.............    3     1     S01
................................ggugagcgcgccgccgucgagG.............    6     1     S01
```

tae-nsmR9

```
cauccccuucgccggcugcgcguccgccuugguggugagcgcgccgccgucgagcgguuuggcugcgg
.................................ggugagcgcgccgcUgucgagc.............   1    1    S01
.................................ggugagcgcgccgcGgucgagc.............   1    1    S01
.................................ggugUgcgcgccgccgucgagc.............   1    1    S01
.................................ggugagcgcgccgccgucgagUg............   4    1    S01
.................................ggugagcgcgccgccgucgaAcg............   1    1    S01
.................................ggugagcgcgccAccgucgagcg............   1    1    S01
.................................ggugagcgcgccgccgucgagcg............   2    0    S01
.................................ggugagcgcgccgccgucgagGg............   5    1    S01
.................................ggugagcgcgccAccgucgagcgg...........   1    1    S01
..................................gugagUgcgccgccgucgag..............   1    1    S01
..................................gugagcgcgccgcUgucgag..............   1    1    S01
..................................gugagcgcgccgccgucgag..............   2    0    S01
..................................gugagcgcgccgccgucgagc.............   1    0    S01
...................................ugagcgcgccgccgucgagc.............   3    0    S01
...................................ugagcgcgccgccgucgagGg............   78   1    S01
...................................ugagcgcAccgccgucgagcg............   1    1    S01
...................................ugagcgcgccgccgucgagcg............   1    0    S01
...................................ugagcgcgccgccgucgaAcg............   17   1    S01
...................................ugagcgcgccAccgucgagcg............   4    1    S01
...................................ugagcgcgccgccgucgagcA............   1    1    S01
.....................................agcUcgccgccgucgagcgg...........   1    1    S01
.....................................agcgcgccAccgucgagcgg...........   2    1    S01
.....................................agcgcgccAccgucgagcggu..........   25   1    S01
.....................................agcgcgccgccgucgaAcggu..........   1    1    S01
.....................................agcgcgccgccgucgagUggu..........   66   1    S01
.....................................agcgcgccgccgucgagcggA..........   6    1    S01
.....................................agcgcgccgcUgucgagcggu..........   8    1    S01
.....................................agcgcgccgccgucgagcggAu.........   1    1    S01
.....................................agcgcgccgccgucgaAcgguu.........   1    1    S01
.....................................agcgcgccgccgucgagUgguu.........   13   1    S01
.....................................agcgcgccAccgucgagcgguu.........   4    1    S01
......................................gcgcgccgccgucgagUgguu.........   1    1    S01
.......................................cgcgccgccgucgagcgguuuggU.....   1    1    S01
.......................................cgcgUcgccgucgagcgguuuggc.....   2    1    S01
.......................................cgcgccgccgucgaAcgguuuggc.....   1    1    S01
..........................................gccgccgucgagcgguuuggc.....   1    0    S01
..............................................ccgucgagcgguuuggcugcg.   1    0    S01
...............................................Ugucgagcgguuuggcugcgg   1    1    S01

cauccccuucgccggcugcg................................................   42   0    S02
cauccccuCcgccggcugcgc...............................................   13   1    S02
cauccccuucUccggcugcgc...............................................   4    1    S02
cauccccuucgcAggcugcgc...............................................   2    1    S02
cauccccuucgccggcugGgc...............................................   1    1    S02
Gauccccuucgccggcugcgc...............................................   5    1    S02
cauccccuuAgccggcugcgc...............................................   1    1    S02
cauccccuucgccggcuUcgc...............................................   3    1    S02
cauAcccuucgccggcugcgc...............................................   1    1    S02
caCcccuucgccggcugcgc................................................   14   1    S02
caucccUuucgccggcugcgc...............................................   6    1    S02
cauccUcuucgccggcugcgc...............................................   6    1    S02
caAccccuucgccggcugcgc...............................................   2    1    S02
cauccccuucgccggcugUgc...............................................   5    1    S02
Aauccccuucgccggcugcgc...............................................   5    1    S02
caucccAucgccggcugcgc................................................   1    1    S02
cauccccuucAccggcugcgc...............................................   5    1    S02
cUuccccuucgccggcugcgc...............................................   7    1    S02
caucAccuucgccggcugcgc...............................................   1    1    S02
Uauccccuucgccggcugcgc...............................................   14   1    S02
cauccccuucgGcggcugcgc...............................................   2    1    S02
cauccccuucgccgAcugcgc...............................................   4    1    S02
cauccAcuucgccggcugcgc...............................................   1    1    S02
caucUccuucgccggcugcgc...............................................   6    1    S02
cauUcccuucgccggcugcgc...............................................   9    1    S02
cauccccuuUgccggcugcgc...............................................   1    1    S02
cauccccuucgccggcGgcgc...............................................   3    1    S02
cauccccuucgccggcugcgU...............................................   23   1    S02
cauccccuucgccAgcugcgc...............................................   4    1    S02
cauccccuucgccggcugcgA...............................................   9    1    S02
cauccccuuGgccggcugcgc...............................................   1    1    S02
```

tae-nsmR9

```
cauccccuucgccggcugcgcguccgccuuguggugagcgcgccgccgucgagcgguuuggcugcgg
cauccccuucgcUggcugcgc..............................................    6       1    S02
cauccccuucgccggAugcgc..............................................    1       1    S02
cauccccuucgUcggcugcgc..............................................    5       1    S02
cauccccuucgccggcugcgc..............................................    5076    0    S02
cauccccuucgccggcugcAc..............................................    2       1    S02
caucccGuucgccggcugcgc..............................................    18      1    S02
cauccccuucgccgUcugcgc..............................................    5       1    S02
cauccccuucgccggcugcgcC.............................................    1       1    S02
cauccccuucgccggcugcgcg.............................................    4       0    S02
cauccccuucgccggcugcgcA.............................................    11      1    S02
cauccccuucgccggcugcgcAu............................................    9       1    S02
cauccccuucgccggcugcgcAuc...........................................    53      1    S02
cauccccuucgccggcugcgcguU...........................................    13      1    S02
.auccccuucgccggcGgcgcg.............................................    1       1    S02
.....cGuucgccggcugcgcguccg.........................................    3       1    S02
.....ccuucgccggcugcgcguUcg.........................................    1       1    S02
......Guucgccggcugcgcguccg.........................................    9       1    S02
......cuucgccggcugcgcguUcg.........................................    8       1    S02
......cuucgccggcugcgcguUcgc........................................    11      1    S02
......cuucgccggcugcgcguUcgcc.......................................    9       1    S02
......cuucgccggcugcgcguUcgccu......................................    16      1    S02
......cuucgccggcugcgcguUcgccuu.....................................    6       1    S02
......cuucgccggcugcgcguUcgccuug....................................    4       1    S02
.......uucgccggcugcgcguccgc........................................    7       0    S02
.......uucgccggcugcgcguUcgcc.......................................    1       1    S02
.......uucgccggcugcgcguccgcc.......................................    2       0    S02
.......uucgccggcugcgcguccgcU.......................................    1       1    S02
.......uucgccggcugcgcguccgccu......................................    1       0    S02
.......uucgccggcugcgcguUcgccu......................................    1       1    S02
.......uucgccggcugcgcguccgcUu......................................    1       1    S02
.......uucgccggcugcgcguUcgccuu.....................................    1       1    S02
.......uucgccggcugcgcguccgccuu.....................................    4       0    S02
.......uucgccggcugcgcguccgccuug....................................    7       0    S02
.......uucgccggcugcgcguccgccuuC....................................    1       1    S02
.......uucgccggcugcgcguccgccuugC...................................    1       1    S02
........ucgccggcugcgcguccAcc.......................................    3       1    S02
........ucgccggcugcgcguccAccu......................................    1       1    S02
........ucgccggcugcgcguccgccu......................................    1       0    S02
..............................guggugagcgcgccgccgucgag..............    1       0    S02
..............................gCggugagcgcgccgccgucgagcg............    1       1    S02
...............................Cggugagcgcgccgccgucgag..............    4       1    S02
...............................Cggugagcgcgccgccgucgagc.............    19      1    S02
...............................uggugagcgcgccgccgucgagc.............    1       0    S02
...............................Aggugagcgcgccgccgucgagc.............    1       1    S02
...............................Cggugagcgcgccgccgucgagcg............    1       1    S02
................................ggugagcgcgccgcUgucga...............    1       1    S02
................................ggugagcgcgccgccgucga...............    4       0    S02
................................ggugagcgcgccgccgucAag..............    1       1    S02
................................ggugagcgcgccgccgucgag..............    6       0    S02
................................ggugUgcgcgccgccgucgag..............    2       1    S02
................................ggugagcgcgcUgccgucgag..............    1       1    S02
................................ggugagcgcgccgccgucgaAc.............    2       1    S02
................................ggugagcgcgccgccgucgagG.............    7       1    S02
................................ggugagcgcgccgccgucgagU.............    2       1    S02
................................ggugagcgcgccgccgucgagc.............    14      0    S02
................................ggugGgcgcgccgccgucgagc.............    1       1    S02
................................ggugagcgcgccgcUgucgagc.............    2       1    S02
................................ggugGgcgcgccgccgucgagcg............    1       1    S02
................................ggugagcgcgccgccgucgagGg............    5       1    S02
................................ggugagcgcgccgccgucgagcg............    1       0    S02
................................ggugagcgcgccAccgucgagcg............    1       1    S02
................................ggugagcgcgccgccgucgagUg............    1       1    S02
................................ggugagcgcgccgcUgucgagcg............    1       1    S02
.................................gugagcgcgccgccgucgag..............    4       0    S02
.................................gugagcgcgccgccgucgagc.............    5       0    S02
.................................gugGgcgcgccgccgucgagc.............    1       1    S02
.................................gugagcgcgccgccgucgagcg............    1       0    S02
.................................gugagcgcgccgccgucgagGg............    3       1    S02
..................................ugGgcgcgccgccgucgagc.............    1       1    S02
..................................ugagcgcgccgccgucgagc.............    2       0    S02
```

tae-nsmR9

caucccuucgccggcugcgcguccgccuuguggugagcgcgccgccgucgagcgguuuggcugcgg			
.................................ugagcgcgccgccgucgagG.............	1	1	S02
.................................ugagcgcgccgccgucgaAcg............	9	1	S02
.................................ugagcgcgccAccgucgagcg............	4	1	S02
.................................ugagcgcgccgccgucgagGg............	45	1	S02
.................................ugagcgcgccgccgucgagcg............	2	0	S02
.................................ugagcgcgccAccgucgagcggu..........	1	1	S02
..................................gagcgcgccgccgucgagcgU...........	1	1	S02
...................................agcgcgccAccgucgagcgg...........	1	1	S02
...................................agcgcgccgccgucgagUggu..........	68	1	S02
...................................agcgcgccgccgucgagcggA..........	2	1	S02
...................................agcgcgccgcUgucgagcggu..........	4	1	S02
...................................agcgcgccAccgucgagcggu..........	26	1	S02
...................................agcgcgccAccgucgagcgguu.........	2	1	S02
...................................agcgcgccgccgucgagUgguu.........	12	1	S02
.....................................cgcgccgcUgucgagcgguuuggc.....	1	1	S02
....cgccgccgucgagcgguuuggc.....	2	0	S02
....cgccgucgagcgguuuggcCgcgg	1	1	S02
....gcUgucgagcgguuuggcugcgg	1	1	S02
....Ugucgagcgguuuggcugcgg	4	1	S02
....cgucgagcgguuuggcugUgg	1	1	S02

miRBase precursor	: tae-nSMR10
Total read count	: 25
tae-nsmR10 read count	: 23
remaining reads	: 2

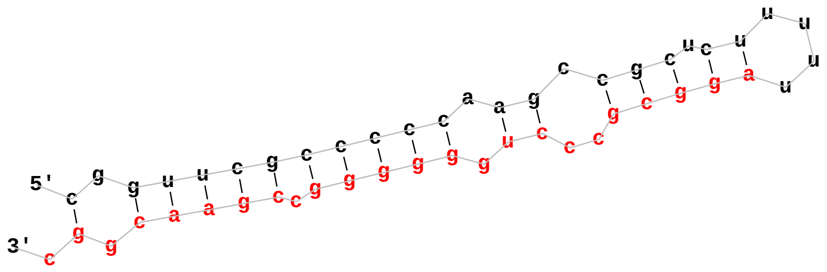

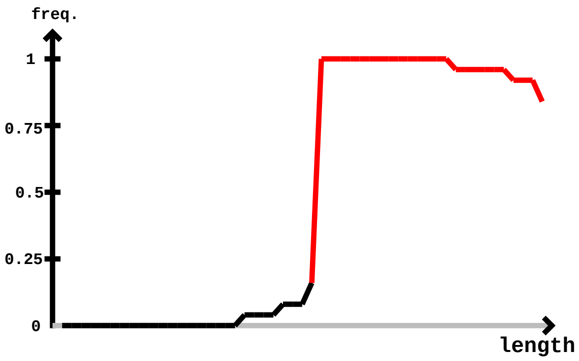


```
                                  tae-nsmR10
5'- cgguucgcccccaagccgcucuuuuuaggcgcccugggggggccgaacggc   -3'    exp
    (.((((((((((.((.(((.((....)))))..)).))))).))))).).    reads      mm         sample
    .........................uUggcgcccugggggggccgaacgg.    1          1          S01
    .........................uaggcgcccugggggggGcgaacgg.    1          1          S01
    ..........................aUgcgcccugggggggccgaacggc    4          1          S01
    ..........................agAcgcccugggggggccgaacggc    4          1          S01
    ..........................aggcgcccugggggggccgaacggc    6          0          S01
    ..........................aggcgcccugggggggccgaacggU    1          1          S01
    ..........................Uggcgcccugggggggccgaacggc    1          1          S01
    ..........................aggcgccUugggggggccgaacggc    1          1          S01
    ..........................aggcgcccugAggggccgaacggc    1          1          S01

    ..................cucuAuuuaggcgcccuggggg..........    1          1          S02
    ......................Auuuaggcgcccugggggggccgaa....    1          1          S02
    ..........................aAgcgcccugggggggccgaacggc    1          1          S02
    ..........................aUgcgcccugggggggccgaacggc    1          1          S02
    ..........................aggcgcccugggggggccgaacggc    1          0          S02
```
